# Treatment effect estimation using the propensity score in clinical trials with historical control

**DOI:** 10.1186/s12874-023-02127-9

**Published:** 2024-02-22

**Authors:** Saki Kanamori, Masahiro Takeuchi

**Affiliations:** 1 Department of Clinical Medicine (Biostatistics), Graduate School of Pharmaceutical Sciences, Kitasato University, 5-9-1, Shirokane, Minato-ku, Tokyo, 108-8641 Japan; 2 Department of Clinical Medicine (Biostatistics), School of Pharmacy, Kitasato University, 5-9-1, Shirokane, Minato-ku, Tokyo, 108-8641 Japan; 3 Graduate School of Mathematical Sciences, The University of Tokyo, 3-8-1, Komaba, Meguro-ku, Tokyo, 153-8914 Japan

**Keywords:** Historical control, Propensity score, Causal inference, Randomized controlled trial, Clinical trial

## Abstract

**Background:**

Clinical trials assessing new treatment effects require a control group to compare the pure treatment effects. However, in clinical trials on regenerative medicine, rare diseases, and intractable diseases, it may be ethically difficult to assign participants to the control group. In recent years, the use of historical control data has attracted attention as a method for supplementing the number of participants in the control group. When combining historical control data with new randomized controlled trial (RCT) data, the assessment of heterogeneity using outcome data is not sufficient. Therefore, several statistical methods that consider participant outcomes and baseline characteristics, including the propensity score (PS) method have been proposed.

**Methods:**

We propose a new method considering “information on whether the data are RCT data or not” in the PS model when combining the RCT and historical control data. The performance of the proposed method in estimating the treatment effect is evaluated using simulation data.

**Results:**

When the distribution of covariates is similar between the RCT and historical control data, not much difference in performance is found between the proposed and conventional methods to estimate the treatment effect. On the other hand, when the distribution of covariates is not similar between the two kinds of data, the proposed method shows higher performance.

**Conclusions:**

Even when it is not known whether RCT and historical control data are similar, the proposed PS model is useful to estimate the treatment effect appropriately in RCTs using historical control data.

**Supplementary Information:**

The online version contains supplementary material available at 10.1186/s12874-023-02127-9.

## Introduction

Clinical trials that assess new treatment effects require a control group to compare the pure treatment effects, which exclude baseline characteristics [1]. Randomized controlled trials (RCTs) are considered the gold standard approach in confirmatory trials for reducing bias and assessing objective effects. However, in clinical trials for regenerative medicine, rare diseases, and intractable diseases, random assignment of participants to the control group may be ethically difficult. Recently, there has been active collection of real-world data and construction of a disease registry [2–4], and the utilization of historical control data has attracted attention as a supplement for the number of control group participants in clinical trials. Appropriate utilization of historical control data can ensure that patients are offered promising treatments faster by reducing the number of participants assigned to control groups, thus accelerating drug development [5, 6]. The U.S. Food and Drug Administration has issued draft guidance on natural history studies for rare disease drug development [7], and further utilization of external control is expected [2–4].

The use of historical control data is still being debated [8–10]. Frequentist approaches include, the pooling method in which historical control data are equated with the new trial control group and merged as is, and the test-then-pool method, which is used after determining the similarity between both outcome data by hypothesis test [11]. Bayesian approaches include power priors [12] and hierarchical modeling [13, 14], which discount the amount of information in historical control data [11, 15]. A previous study proposed a method that calculates the difference between outcome data of a new trial control group and historical control data and used weighting as an estimate of heterogeneity [16]. Evaluation of heterogeneity with outcome data is useful, but not sufficient in situations with different measurement periods and conditions. Besides, the information from historical control data may distort the true results from new trials [15], or conversely, historical control data may be hardly used [16], which poses a large risk for implementation.

In the causal inference framework, propensity scores (PS) [17, 18] may be used to compare groups that are not randomized. The PS indicates the probability of treatment allocation calculated using baseline characteristics. Thus, by aligning the baseline characteristics between treatment groups, it is possible to estimate the treatment effect while minimizing the effect of confounding on treatment allocation. When utilizing historical control data, a method using the PS has been proposed for considering the heterogeneity of baseline characteristics. In general, the matching [19, 20] and inverse probability of treatment weighting (IPTW) [21] methods are used as PS methods [22, 23]. Methods using PS to assess the generalizability of the population participating in RCT to the patient population [24], and to merge RCT data with observational data [25] have also been proposed. Additionally, a method combining the PS methods and Bayesian dynamic borrowing framework has been proposed [26].

Furthermore, as this study considers a special clinical trial that uses historical data in combination with new RCT data includes information on whether the data are RCT or historical control data. This information could be an important confounding factor along with baseline characteristics. Accordingly, we evaluate the performance of the method used for the clinical trial that newly considers “information on whether the data are RCT data or not” in the conventional PS model when estimating the treatment effect using simulation data.

## Proposal of the PS model

In a clinical trial in which the primary endpoint is binary outcome $Y$ (presence or absence of an event), we assume historical control data are combined with new two-armed RCT data as part of a control group. ${Y}_{i}=1$ indicates that an event has occurred, and ${Y}_{i}=0$ indicates that no event has occurred with participant $i\ \left(i=1\dots l\right)$. We set $T$ as the treatment group indicator (${T}_{i}=1$ for the treatment and ${T}_{i}=0$ for the control groups for participant $i$) and $X$ as the vector of all covariates ${X}_{j}$ ($j=1\dots\textit{k}$ and ${X}_{ij}$ denotes the $\textit{j}$th covariate of participant $i$), which are the possible confounding factors. When estimating the PS, a model would generally be expressed as

1 $$\begin{array}{c}\pi =\mathrm{logit}\left\{{\text{Pr}}\left(T=1|X\right)\right\}={\beta }_{0}+{\beta }_{1}{X}_{1}+{\beta }_{2}{X}_{2}+\dots +{\beta }_{k}{X}_{k},\end{array}$$

using a logistic regression [27, 28], where $\beta_j\ \left(j=1\dots\textit{k}\right)$ denotes a coefficient of the regression model.

Here, we might consider the information on whether the data were derived from the new RCT or historical control data as an important confounding factor. Therefore, in the proposed method of this study, the PS model newly considers information on whether the data are RCT data or not and sets that information as indicator variable $X_\textit{r}$. ${X}_{ir}=1$ indicates that participant $i$ is from the RCT, and ${X}_{ir}=-1$ indicates that participant $i$ is from the historical control group. As a proposed method including $X_\textit{r}$, the PS model could be expressed as

2 $$\begin{array}{c}{\pi }^{*}=\mathrm{logit}\left\{{\text{Pr}}\left(T=1|X,{X}_{r}\right)\right\}={\beta }_{0}+{\beta }_{1}{X}_{1}+{\beta }_{2}{X}_{2}+\dots +{\beta }_{k}{X}_{k}+{\beta }_{r}{X}_{r}.\end{array}$$

We considered that the performance in estimating the treatment effect between the conventional method using $\pi$ and proposed method using ${\pi }^{*}$ may vary due to the difference in the distribution of covariates between the RCT and historical control data. In Stimulation study section, we evaluate the performance of the method using simulated data.

As a PS method, although the matching method is easy to understand, there is a possibility that the amount of information will be drastically reduced. In this study, we apply the IPTW method to utilize more information when evaluating the model’s performance. When estimating the treatment effect, each participant’s weight $w$ could be $w=T/{\text{expit}}\left(\pi \right)+\left(1-T\right)/\left\{1-{\text{expit}}\left(\pi \right)\right\}$ in the conventional method and $w=T/{\text{expit}}\left({\pi }^{*}\right)+\left(1-T\right)/\left\{1-{\text{expit}}\left({\pi }^{*}\right)\right\}$ in the proposed method.

## Simulation study

### Settings

In this simulation study, to evaluate the treatment effect, we set the total number of participants as *n* = 900 and the allocation ratio between the RCT treatment group, RCT control group, and historical control group as 1:1:2. Moreover, we set the outcome event rates as 50%, 10%, and 5%; the odds ratios as 1.0, 2.0, 5.0, and 10.0; and the two-sided significance level as 5%. Furthermore, we also examined cases where the number of participants was small. The simulation results assuming the total number of participants as *n* = 200 are shown. The method and conditions in the simulation setting are the same as those shown in the setting assuming that *n* = 900, except for the total number of participants. The supplementary examination was conducted by assuming a situation with odds ratios of 1.5 and 2.5 (Additional file 1: Appendix A). In addition, we assume a situation wherein the allocation ratios are different (Additional file 1: Appendix B) and one of the four covariates is binary data (Additional file 1: Appendix C). We also conducted simulations in which the assignment of treatment variables was completely random in the RCT population (Additional file 1: Appendix D), and simulations were based on parameter settings from the actual clinical trial [29] (Additional file 1: Appendix G). To estimate the treatment effect, the IPTW using the PS method is applied, and the odds ratio based on the weight is estimated by the logistic regression model.

The performance measurements of the simulation result include the following: (1) difference of the estimated log odds ratio from the true log odds ratio (bias), (2) mean squared error (MSE), (3) coverage of 95% confidence interval (coverage), and (4) type I error rate and power. The simulation data are generated while assuming two scenarios wherein the distribution of covariates is either similar or not similar between the RCT and historical control data.

#### Scenario (I)

In this situation, the distribution of covariates is similar between the RCT and historical control data. From the multivariate standard normal distribution, four covariates are generated for participant $i$ as

3 $$\begin{array}{c}\left\{X_{i1},X_{i2},X_{i3},X_{i4}\right\}\sim N\left(0,1\right)\end{array}.$$

Here, the true PS model $\pi_{\textit{i},\textit{true}}$ is

4 $$\begin{array}{c}\pi_{\textit{i},\textit{true}}=\mathrm{logit}\left\{\text{Pr}\left(T=1\vert X\right)\right\}=\beta_0+\beta_1X_{i1}+\beta_2X_{i2}+\beta_3X_{i3}+\beta_4X_{i4},\end{array}$$

and the parameters are $\left\{{\beta }_{0}, { \beta }_{1}, { \beta }_{2},{ \beta }_{3},{ \beta }_{4}\right\}=\left\{{b}_{0}, 1.00, -0.50, 0.25, 0.10\right\}$. ${b}_{0}$ is a constant correction value corresponding to the treatment allocation ratio (Additional file 1: Appendix E). Based on Eq. (4), each participant’s treatment allocation is determined from the Bernoulli distribution:

5 $$\begin{array}{c}T_i\sim Bernoulli\left\{\frac{\text{exp}\left(\pi_{\textit{i},\textit{true}}\right)}{1+\text{exp}\left(\pi_{\textit{i},\textit{true}}\right)}\right\}.\end{array}$$

The model that generates outcome data ${y}_{i}$ is as follows:

6 $$\begin{array}{c}y_\textit{i}=\mathrm{logit}\left\{\text{Pr}\left(Y=1\vert X\right)\right\}=\alpha_0+\beta_{treat}T_i+\alpha_1X_{i1}+{\alpha_2X}_{i2}+{\alpha_3X}_{i3}+\alpha_4X_{i4}+\varepsilon_i/100,\end{array}$$

where $\left\{{\alpha }_{0},{ \alpha }_{1},{ \alpha }_{2},{ \alpha }_{3},{ \alpha }_{4}\right\}=\left\{{a}_{0}, 0.274, 0.137, -0.137, 0.137\right\}$. Here, ${\beta }_{treat}$ is the true log odds ratio of the treatment effect, and the error term ${\varepsilon }_{i}\sim N\left(0, 1\right)$ is generated according to independent normal distribution. Besides, ${a}_{0}$ is a constant correction value corresponding to the outcome event rate (Additional file 1: Appendix E). Based on Eq. (6), each participant’s outcome ${Y}_{i}$ is determined from the Bernoulli distribution:

7 $$\begin{array}{c}Y_i\sim Bernoulli\left\{\frac{\text{exp}\left(y_\textit{i}\right)}{1+\text{exp}\left(y_\textit{i}\right)}\right\}.\end{array}$$

#### Scenario (II)

In this situation, the distribution of covariates is not similar between the RCT and historical control data. As with scenario (I), after generating covariates from the multivariate standard normal distribution,

8 $$\begin{array}{c}\left\{{{X}^\prime}_{i1},{ {X}^\prime}_{i2},{ {X}^\prime}_{i3},{ {X}^\prime}_{i4}\right\}\sim N\left(0, 1\right),\end{array}$$

each covariate in the RCT data are transformed as follows:

9 $$\begin{array}{c}\left\{{X}_{i1}={{X}^\prime}_{i1}-1, { X}_{i2}={ {X}^\prime}_{i2}\times 0.7, { X}_{i3}={\text{ln}}\left|{{X}^\prime}_{i3}\right|,{ X}_{i4}={ {X}^\prime}_{i4}\right\}.\end{array}$$

For historical control data, the covariates without transformation, ${X}_{i1},{ X}_{i2},{ X}_{i3},\mathrm{and}\, {X}_{i4}$, are simply used from the generation of standard multivariate normal distributions, that is,

10 $$\begin{array}{c}\left\{{X}_{i1}={{X}^\prime}_{i1}, { X}_{i2}={ {X}^\prime}_{i2}, { X}_{i3}={{X}^\prime}_{i3},{ X}_{i4}={ {X}^\prime}_{i4}\right\}.\end{array}$$

Here, the true PS model $\pi_{\textit{i},\textit{true}}^{\mathit\ast}$ is provided as

11 $$\begin{array}{c}\pi_{\textit{i},\textit{true}}^{\mathit\ast}=\mathrm{logit}\left\{\text{Pr}\left(T=1\vert X,X_r\right)\right\}=\beta_0+{\beta_1X}_{i1}+{\beta_2X}_{i2}+{\beta_3X}_{i3}+{\beta_4X}_{i4}+{\beta_rX}_{ir},\end{array}$$

where $\left\{{\beta }_{0}, { \beta }_{1}, { \beta }_{2},{ \beta }_{3},{ \beta }_{4},{ \beta }_{r}\right\}=\left\{\left({b}_{0}-{b}_{r}\right), 1.00, -0.50, 0.25, 0.10, {b}_{r}\right\}$. ${b}_{r}$ is the coefficient value of indicator variable ${X}_{r}$ in the true PS model for each treatment allocation ratio (Additional file 1: Appendix F). These parameters are simultaneously calculated using a true PS model for only RCT data,

12 $$\begin{array}{c}\pi_{\textit{i},\textit{true},\textit{RCT}}=\mathrm{logit}\left\{\text{Pr}\left(T=1\vert X\right)\right\}=b_0+{1.00X}_{i1}-{0.50X}_{i2}+{0.25X}_{i3}+{0.10X}_{i4};\end{array}$$

the true PS model for only historical control data,

13 $$\begin{array}{c}\pi_{\textit{i},\textit{true},\textit{HC}}=\mathrm{logit}\left\{\text{Pr}\left(T=1\right)\right\}=0;\end{array}$$

and a covariate of each participant (Additional file 1: Appendix F; calculation method). Based on Eq. (12), treatment allocation ${T}_{i}$ for each participant is determined from the Bernoulli distribution:

14 $$\begin{array}{c}T_i\sim Bernoulli\left\{\frac{\text{exp}\left(\pi_{\textit{i},\textit{true}}^{\mathit\ast}\right)}{1+\text{exp}\left(\pi_{\textit{i},\textit{true}}^{\mathit\ast}\right)}\right\}.\end{array}$$

Outcome data ${y}_{i}$ are generated by

15 $$\begin{array}{c}y_\textit{i}=\mathrm{logit}\left\{\text{Pr}\left(Y=1\vert X\right)\right\}=\alpha_0+\beta_{treat}T_i+\alpha_1X_{i1}+\alpha_2X_{i2}+\alpha_3X_{i3}+\alpha_4X_{i4}+\alpha_rX_{ir}+\varepsilon_i/100,\end{array}$$

where $\left\{{\alpha }_{0},{ \alpha }_{1},{ \alpha }_{2},{ \alpha }_{3},{ \alpha }_{4},{ \alpha }_{r}\right\}=\left\{{a}_{0},0.274, 0.137, -0.137, 0.137, 0.137\right\}$. Based on Eq. (15), each participant’s outcome ${Y}_{i}$ is determined from the Bernoulli distribution:

16 $$\begin{array}{c}Y_i\sim Bernoulli\left\{\frac{\text{exp}\left(y_\textit{i}\right)}{1+\text{exp}\left(y_\textit{i}\right)}\right\}.\end{array}$$

### Results

#### The usual number of participants

In scenario (I), wherein the distribution of covariates is similar between the RCT and historical control data, not much difference in the proposed and conventional methods was found in the bias, MSE, coverage of 95% confidence interval, and type I error (Table 1).

**Table 1:** Scenario (I): performance of the estimated propensity score (PS) model

Performance measurement	PS model	Outcome event rate	Odds ratio			
			1.0	2.0	5.0	10.0
Bias	$\pi$ (without $X_\textit{r}$)	50%	0.004	-0.016	-0.034	-0.032
		10%	-0.026	-0.022	-0.018	-0.013
		5%	-0.060	-0.031	-0.009	0.017
	${\pi }^{*}$ (with $X_\textit{r}$)	50%	0.035	0.014	-0.005	-0.007
		10%	0.019	0.020	0.023	0.030
		5%	0.000	0.023	0.045	0.074
MSE	$\pi$	50%	0.045	0.050	0.069	0.097
		10%	0.117	0.092	0.086	0.097
		5%	0.228	0.175	0.160	0.201
	${\pi }^{*}$	50%	0.037	0.039	0.052	0.074
		10%	0.097	0.080	0.084	0.100
		5%	0.187	0.154	0.160	0.217
Coverage (%)	$\pi$	50%	95.0	94.6	94.0	93.2
		10%	93.9	94.3	94.8	94.7
		5%	93.2	93.9	94.3	94.4
	${\pi }^{*}$	50%	94.9	94.8	94.8	94.3
		10%	94.7	94.9	94.6	94.4
		5%	94.3	94.1	94.5	94.1
Type I error and power (%)	$\pi$	50%	5.0	86.6	99.8	100.0
		10%	6.1	63.7	100.0	100.0
		5%	6.8	41.7	98.5	100.0
	${\pi }^{*}$	50%	5.0	94.1	99.9	100.0
		10%	5.2	72.4	99.8	100.0
		5%	5.7	50.7	98.4	99.8

$\pi$ (without $X_\textit{r}$): the conventional method; ${\pi }^{*}$ (with $X_\textit{r}$): the proposed method

On the other hand, in scenario (II), wherein the distribution of covariates is not similar between the RCT and historical control data, the proposed method tended to have a smaller bias, coverage of 95% confidence interval closer to 95%, and a type I error rate closer to 5%. In addition, there was not much difference between the proposed and conventional methods in the MSE (Table 2).

**Table 2:** Scenario (II): performance of the estimated propensity score (PS) model

Performance measurement	PS model	Outcome event rate	Odds ratio			
			1.0	2.0	5.0	10.0
Bias	$\pi$ (without $X_\textit{r}$)	50%	0.169	0.151	0.135	0.128
		10%	0.154	0.155	0.151	0.151
		5%	0.135	0.153	0.172	0.190
	${\pi }^{*}$ (with $X_\textit{r}$)	50%	0.044	0.026	0.010	0.006
		10%	0.029	0.034	0.035	0.038
		5%	0.007	0.032	0.062	0.091
MSE	$\pi$	50%	0.053	0.049	0.052	0.065
		10%	0.090	0.079	0.080	0.092
		5%	0.154	0.132	0.140	0.212
	${\pi }^{*}$	50%	0.036	0.038	0.050	0.070
		10%	0.094	0.080	0.084	0.103
		5%	0.183	0.150	0.161	0.248
Coverage (%)	$\pi$	50%	81.5	85.6	90.4	92.7
		10%	89.6	89.6	90.6	92.6
		5%	91.8	92.1	93.2	94.9
	${\pi }^{*}$	50%	94.4	95.1	94.7	94.3
		10%	94.5	94.1	94.1	94.1
		5%	94.1	94.2	93.7	93.8
Type I error and power (%)	$\pi$	50%	18.5	99.9	100.0	100.0
		10%	10.4	94.2	100.0	100.0
		5%	8.2	74.7	100.0	100.0
	${\pi }^{*}$	50%	5.6	96.1	100.0	100.0
		10%	5.5	74.9	99.9	100.0
		5%	5.8	52.5	98.8	99.9

$\pi$ (without $X_\textit{r}$): the conventional method, ${\pi }^{*}$ (with $X_\textit{r}$): the proposed method

When the allocation ratios between the RCT treatment group, RCT control group, and historical control group were 2:1:3 (Additional file 1: Appendix Table B.3), 1:1:4 (Additional file 1: Appendix Table B.5), 2:1:6 (Additional file 1: Appendix Table B.6), 2:1:1 (Additional file 1: Appendix Table B.11), and 3:1:2 (Additional file 1: Appendix Table B.12)—that is, different but not extremely skewed—the same tendency in all performance measurements was observed as in the allocation ratio of 1:1:2. However, when the allocation ratios were 9:1:10 (Additional file 1: Appendix Table B.4), 9:1:20 (Additional file 1: Appendix Table B.7), 1:1:18 (Additional file 1: Appendix Table B.8), 2:1:27 (Additional file 1: Appendix Table B.9), and 9:1:90 (Additional file 1: Appendix Table B.10)—that is, extremely skewed—the bias and MSE had increased.

In addition, the same trends were observed for all performance measures when one of the four covariates was binary data (Additional file 1: Appendix Table C.1) as when the four covariates were continuous data.

Moreover, the simulation where the treatment variable in RCT population was generated independent of covariates (Additional file 1: Appendix Table D.1) shown also almost the same result in the text. In a simulation where the parameter settings of an actual clinical trial were applied (Additional file 1: Appendix Table G.1) was also similar result in the text.

#### Small number of participants

In the case where the total number of participants is *n* = 200, the same tendency was observed in all performance measurements as in the case where the number of participants is *n* = 900.

That is, in scenario (I), wherein the distribution of covariates is similar between the RCT and historical control data, not much difference in the proposed and conventional methods was found in the bias, MSE, coverage of 95% confidence interval, and type I error rate (Table 3).

**Table 3:** Scenario (I): performance of the estimated propensity score (PS) model by simulation setting assuming *n* = 200

Performance measurement	PS model	Outcome event rate	Odds ratio			
			1.0	2.0	5.0	10.0
Bias	$\pi$ (without $X_\textit{r}$)	50%	-0.001	0.002	0.034	0.112
		10%	-0.199	-0.076	-0.007	0.104
		5%	-1.264	-0.356	0.312	1.424
	${\pi }^{*}$ (with $X_\textit{r}$)	50%	0.030	0.029	0.048	0.105
		10%	-0.101	0.006	0.069	0.187
		5%	-1.087	-0.218	0.447	1.593
MSE	$\pi$	50%	0.229	0.245	0.336	0.865
		10%	1.835	0.555	0.611	1.618
		5%	22.016	7.358	7.516	25.242
	${\pi }^{*}$	50%	0.187	0.202	0.271	0.711
		10%	1.636	0.493	0.605	1.702
		5%	20.447	6.941	8.031	27.171
Coverage (%)	$\pi$	50%	93.3	93.2	92.5	90.6
		10%	91.8	92.5	93.2	93.9
		5%	88.4	91.2	92.1	85.8
	${\pi }^{*}$	50%	94.2	94.4	93.8	93.4
		10%	93.6	93.2	93.2	93.6
		5%	89.3	92.8	92.9	87.2
Type I error and power (%)	$\pi$	50%	6.5	35.7	88.0	96.7
		10%	8.1	21.5	74.9	94.7
		5%	11.4	16.7	51.9	78.2
	${\pi }^{*}$	50%	5.6	41.2	92.8	98.1
		10%	6.1	26.4	79.3	95.5
		5%	10.6	21.0	60.3	83.1

$\pi$ (without $X_\textit{r}$): the conventional method; ${\pi }^{*}$ (with $X_\textit{r}$): the proposed method

And then, in scenario (II), wherein the distribution of covariates is not similar between the RCT and historical control data, the proposed method tended to have a smaller bias, coverage of 95% confidence interval closer to 95%, and a type I error rate closer to 5%. In addition, there was not much difference between the proposed and conventional methods in the MSE (Table 4).

**Table 4:** Scenario (II): performance of the estimated propensity score (PS) model by simulation setting assuming *n* = 200

Performance measurement	PS model	Outcome event rate	Odds ratio			
			1.0	2.0	5.0	10.0
Bias	$\pi$ (without $X_\textit{r}$)	50%	0.168	0.164	0.168	0.224
		10%	0.032	0.132	0.177	0.268
		5%	-0.908	-0.106	0.516	1.758
	${\pi }^{*}$ (with ${X}_{{\text{r}}}$)	50%	0.050	0.050	0.060	0.126
		10%	-0.096	0.011	0.077	0.187
		5%	-0.989	-0.203	0.447	1.750
MSE	$\pi$	50%	0.144	0.151	0.202	0.862
		10%	1.686	0.459	0.326	1.431
		5%	19.574	6.274	7.605	27.879
	${\pi }^{*}$	50%	0.174	0.193	0.261	0.880
		10%	1.640	0.532	0.421	1.584
		5%	18.505	6.120	8.210	29.796
Coverage (%)	$\pi$	50%	92.4	94.0	95.2	95.8
		10%	93.9	94.2	95.1	96.6
		5%	89.8	94.6	95.5	88.4
	${\pi }^{*}$	50%	94.5	94.0	93.6	94.0
		10%	94.0	94.0	93.9	93.7
		5%	90.0	93.4	93.4	86.8
Type I error and power (%)	$\pi$	50%	7.5	68.6	99.9	100.0
		10%	6.0	39.1	93.1	99.5
		5%	10.2	25.2	72.3	92.3
	${\pi }^{*}$	50%	5.4	44.4	94.1	98.7
		10%	5.9	26.6	81.1	96.2
		5%	9.9	20.7	98.8	99.9

$\pi$ (without $X_\textit{r}$): the conventional method, ${\pi }^{*}$ (with $X_\textit{r}$): the proposed method

## Discussion

The results in this study suggest that a situation wherein the distribution of covariates is similar between the RCT and historical control data—that is, scenario (I)—the estimation bias of the treatment effect in the PS model would not be affected by including the information on whether the participant data is RCT data or not. On the other hand, a situation wherein the distribution of covariates is not similar between the RCT and historical control data—that is, scenario (II)—the use of the proposed PS method is recommended because the performance of estimating the treatment effect is improved by including the information on whether the participant data is RCT data or not.

As for the relationship between the outcome event rate and performance of estimating the treatment effect, it is considered appropriate that the higher the outcome event rate, the higher the performance of the estimation. Therefore, in the situation where the distributions of covariates are similar, the treatment effect could be estimated appropriately using both the proposed and conventional methods for this situation. Meanwhile, where the distributions of covariates are not similar, a similar tendency is observed when using the proposed method, and so it is considered that the appropriate treatment effect can be estimated. However, in the conventional method, the lower the outcome event rate, the higher the performance that can be estimated, and so there is a possibility that the appropriate treatment effect cannot be estimated.

Moreover, even when the allocation ratio between the RCT treatment group, RCT control group, and historical control group is changed, if the allocation ratio is not extremely skewed, the same consideration is possible as in the allocation ratio of 1:1:2 in this situation. Namely, in the situation where the distributions of covariates are similar, when considering the information on whether the data are RCT data or not in the PS model, the effect on the performance of estimating the treatment effect was not as marked. And also, in the situation where the distributions of covariates are not similar, the performance of estimating the treatment effect was improved by considering whether the data are RCT data or not. Meanwhile, when the allocation ratio was extremely skewed, bias and MSE increased tremendously, and the estimation could not be conducted appropriately. This is because the number of participants in the RCT control group was extremely small when the allocation ratio was extremely biased.

As another situation, even if the total number of participants is small or and the covariates include binary data, the same consideration is possible as that when the total number of participant is *n* = 900 and the covariates are all continuous data. The same trend is suggested when the treatment variables in the RCT population are considered completely independently and randomly from the covariates. In other words, when the distribution of covariates is similar between the RCT and historical control data, not much difference in performance is found between the proposed and conventional methods to estimate the treatment effect. And, when the distribution of covariates is not similar between the two kinds of data, the proposed method shows higher performance. In addition, the same argument as above can be considered to apply even when there is variation in data such as actual clinical trial data.

For these reasons, when combining the RCT and historical control data in the clinical trial setting, it is important to consider whether the distribution of important participant baseline characteristics that influence the outcomes is similar or not. Moreover, for appropriate utilization of historical control data, it is useful to apply the proposed PS model that considers $X_\textit{r}$ while assessing possible differences. However, when considering the utilization of historical control data to reinforce the number of participants in the RCT control group, it is necessary to simulate several patterns of allocation ratio and evaluate the performance of the allowable range of how small the control group can be from the planning stage of the clinical trial, and use this with caution. In addition, since the proposed method uses PS, the possibility of the presence of unmeasured confounding factors, that is, whether the covariates used in the PS model are sufficient, should also be considered. And, this method is assuming that use single historical control data set, and have limited that could not have considered for difference between two or more historical control data set. Furthermore, in this study, we focused on the treatment effect in the entire population, including historical control data, and investigated a method for estimating the Average Treatment Effect (ATE). However, there may be situations in which it is desirable to estimate the Average Treatment Effect on the Treated (ATT) in the RCT population or treatment group, and we would like to consider the performance evaluation in such cases to be a future issue. While paying attention to issues such as the increase in type I error rate, it is possible to appropriately reduce the number of participants assigned to the RCT control group. We believe that this will help improve the efficiency of clinical trials, solve ethical problems, and thus save more people.

## Conclusions

In clinical trials utilizing historical control data, considering information on whether the data are RCT data or not in the proposed PS model is useful for appropriately estimating the treatment effect, even when it is not known whether the RCT data and the historical control data are similar. Promotion of appropriate utilization of historical control data will contribute to the realization of better medical care.

### Supplementary Information

**Additional file 1:** **Appendix A.** Simulation setting assuming odds ratios 1.5 and 2.5. **Appendix B.** Simulation setting assuming that the allocation ratio between the RCT treatment group, RCT control group, and historical control data is other than 1:1:2. **Appendix C.** Simulation setting assuming that one of the covariates is binary data. **Appendix D.** Simulation setting assuming that the randomized assignment of treatment variables. **Appendix E.** Probability of treatment allocation correction value *b*_0_ and outcome event rate correction value *a*_0_. **Appendix F.** Calculation method of the true PS model in scenario (II). **Appendix G.** Simulation based on actual clinical trial parameter settings.

## Data Availability

The datasets used and/or analysed during the current study are available from the corresponding author on reasonable request.

## Abbreviations

RCT: Randomized controlled trial
PS: Propensity score
IPTW: Inverse probability of treatment weighting
MSE: Mean squared error
Coverage: Coverage of 95% confidence interval
