# Supplementary material for: Treatment effect estimation using the propensity score in clinical trials with historical control
Source: BMC Med Res Methodol. 2024 Feb 22;24:47. doi: 10.1186/s12874-023-02127-9 (PMC10882803; doi:10.1186/s12874-023-02127-9)
Supplement: Supplementary file 1 — Additional file 1: Appendix A. Simulation setting assuming odds ratios 1.5 and 2.5. Appendix B. Simulation setting assuming that the allocation ratio between the RCT treatment group, RCT control group, and historical control data is other than 1:1:2. Appendix C. Simulation setting assuming that one of the covariates is binary data. Appendix D. Simulation setting assuming that the randomized assignment of treatment variables. Appendix E. Probability of treatment allocation correction value b0 and outcome event rate correction value a0. Appendix F. Calculation method of the true PS model in scenario (II). Appendix G. Simulation based on actual clinical trial parameter settings. [file 12874_2023_2127_MOESM1_ESM.docx]

1. **Simulation setting assuming odds ratios 1.5 and 2.5**

In addition to the odds ratios (1.0, 2.0, 5.0, 10.0) dealt with in this study, the expanded simulation results assuming odds ratios 1.5 and 2.5 are shown below.

Performance of the estimated propensity score (PS) model by simulation setting assuming odds ratio (1.0, 1.5, 2.0, 2.5, 5.0, 10.0)

|  |  |  | **Scenario Ⅰ** | | | | | |  | **Scenario Ⅱ** | | | | | |
| --- | --- | --- | --- | --- | --- | --- | --- | --- | --- | --- | --- | --- | --- | --- | --- |
|  |  |  | Odds ratio | | | | | |  | Odds ratio | | | | | |
| Performance measurement | PS model | Outcome event rate | 1.0 | 1.5 | 2.0 | 2.5 | 5.0 | 10.0 |  | 1.0 | 1.5 | 2.0 | 2.5 | 5.0 | 10.0 |
| Bias | $\pi$ | 50% | 0.004 | -0.007 | -0.016 | -0.023 | -0.034 | -0.032 |  | 0.169 | 0.159 | 0.151 | 0.148 | 0.135 | 0.128 |
|  | (without $X_{r}$) | 10% | -0.026 | -0.023 | -0.022 | -0.021 | -0.018 | -0.013 |  | 0.154 | 0.156 | 0.155 | 0.154 | 0.151 | 0.151 |
|  |  | 5% | -0.060 | -0.041 | -0.031 | -0.024 | -0.009 | 0.017 |  | 0.135 | 0.147 | 0.153 | 0.157 | 0.172 | 0.190 |
|  | $\pi^{*}$ | 50% | 0.035 | 0.022 | 0.014 | 0.007 | -0.005 | -0.007 |  | 0.044 | 0.034 | 0.026 | 0.023 | 0.010 | 0.006 |
|  | (with $X_{r}$) | 10% | 0.019 | 0.020 | 0.020 | 0.021 | 0.023 | 0.030 |  | 0.029 | 0.034 | 0.034 | 0.034 | 0.035 | 0.038 |
|  |  | 5% | 0.000 | 0.016 | 0.023 | 0.030 | 0.045 | 0.074 |  | 0.007 | 0.024 | 0.032 | 0.039 | 0.062 | 0.091 |
| MSE | $\pi$ | 50% | 0.045 | 0.047 | 0.050 | 0.052 | 0.069 | 0.097 |  | 0.053 | 0.050 | 0.049 | 0.049 | 0.052 | 0.065 |
|  |  | 10% | 0.117 | 0.099 | 0.092 | 0.090 | 0.086 | 0.097 |  | 0.090 | 0.083 | 0.079 | 0.078 | 0.080 | 0.092 |
|  |  | 5% | 0.228 | 0.193 | 0.175 | 0.166 | 0.160 | 0.201 |  | 0.154 | 0.137 | 0.132 | 0.130 | 0.140 | 0.212 |
|  | $\pi^{*}$ | 50% | 0.037 | 0.038 | 0.039 | 0.041 | 0.052 | 0.074 |  | 0.036 | 0.036 | 0.038 | 0.040 | 0.050 | 0.070 |
|  |  | 10% | 0.097 | 0.085 | 0.080 | 0.080 | 0.084 | 0.100 |  | 0.094 | 0.084 | 0.080 | 0.079 | 0.084 | 0.103 |
|  |  | 5% | 0.187 | 0.165 | 0.154 | 0.150 | 0.160 | 0.217 |  | 0.183 | 0.158 | 0.150 | 0.148 | 0.161 | 0.248 |
| Coverage | $\pi$ | 50% | 95.0 | 94.8 | 94.6 | 94.6 | 94.0 | 93.2 |  | 81.5 | 83.8 | 85.6 | 86.9 | 90.4 | 92.7 |
| (%) |  | 10% | 93.9 | 94.3 | 94.3 | 94.0 | 94.8 | 94.7 |  | 89.6 | 89.6 | 89.6 | 89.5 | 90.6 | 92.6 |
|  |  | 5% | 93.2 | 93.1 | 93.9 | 93.9 | 94.3 | 94.4 |  | 91.8 | 91.8 | 92.1 | 92.1 | 93.2 | 94.9 |
|  | $\pi^{*}$ | 50% | 94.9 | 94.8 | 94.8 | 94.9 | 94.8 | 94.3 |  | 94.4 | 94.7 | 95.1 | 94.8 | 94.7 | 94.3 |
|  |  | 10% | 94.7 | 95.0 | 94.9 | 94.7 | 94.6 | 94.4 |  | 94.5 | 94.2 | 94.1 | 94.2 | 94.1 | 94.1 |
|  |  | 5% | 94.3 | 93.8 | 94.1 | 94.4 | 94.5 | 94.1 |  | 94.1 | 94.1 | 94.2 | 94.5 | 93.7 | 93.8 |
| Type I error | $\pi$ | 50% | 5.0 | 47.2 | 86.6 | 96.3 | 99.8 | 100.0 |  | 18.5 | 95.0 | 99.9 | 100.0 | 100.0 | 100.0 |
| and power |  | 10% | 6.1 | 25.6 | 63.7 | 87.3 | 100.0 | 100.0 |  | 10.4 | 64.4 | 94.2 | 99.3 | 100.0 | 100.0 |
| (%) |  | 5% | 6.8 | 17.4 | 41.7 | 65.8 | 98.5 | 100.0 |  | 8.2 | 41.2 | 74.7 | 90.7 | 100.0 | 100.0 |
|  | $\pi^{*}$ | 50% | 5.0 | 60.0 | 94.1 | 98.9 | 99.9 | 100.0 |  | 5.6 | 64.6 | 96.1 | 99.5 | 100.0 | 100.0 |
|  |  | 10% | 5.2 | 34.0 | 72.4 | 91.4 | 99.8 | 100.0 |  | 5.5 | 36.8 | 74.9 | 92.6 | 99.9 | 100.0 |
|  |  | 5% | 5.7 | 23.1 | 50.7 | 72.4 | 98.4 | 99.8 |  | 5.8 | 23.6 | 52.5 | 73.9 | 98.8 | 99.9 |

[Note] Scenario I: the situation wherein the covariate distribution is similar between the randomized control trial (RCT) data and historical control data; Scenario II: the situation wherein the covariate distribution is not similar between the RCT data and historical control data; $\pi$ (without $X_{r}$): the conventional method; $\pi^{*}$ (with $X_{r}$): the proposed method.

Performance of the estimated propensity score (PS) model by simulation setting assuming odds ratio (1.0, 1.5, 2.0, 2.5, 5.0, 10.0) and $n=200$

|  |  |  | **Scenario Ⅰ** | | | | | |  | **Scenario Ⅱ** | | | | | |
| --- | --- | --- | --- | --- | --- | --- | --- | --- | --- | --- | --- | --- | --- | --- | --- |
|  |  |  | Odds ratio | | | | | |  | Odds ratio | | | | | |
| Performance measurement | PS model | Outcome event rate | 1.0 | 1.5 | 2.0 | 2.5 | 5.0 | 10.0 |  | 1.0 | 1.5 | 2.0 | 2.5 | 5.0 | 10.0 |
| Bias | $\pi$ | 50% | -0.001 | 0.004 | 0.002 | 0.008 | 0.034 | 0.112 |  | 0.168 | 0.160 | 0.164 | 0.165 | 0.168 | 0.224 |
|  | (without $X_{r}$) | 10% | -0.199 | -0.105 | -0.076 | -0.058 | -0.007 | 0.104 |  | 0.032 | 0.103 | 0.132 | 0.147 | 0.177 | 0.268 |
|  |  | 5% | -1.264 | -0.654 | -0.356 | -0.186 | 0.312 | 1.424 |  | -0.908 | -0.334 | -0.106 | 0.040 | 0.516 | 1.758 |
|  | $\pi^{*}$ | 50% | 0.030 | 0.031 | 0.029 | 0.032 | 0.048 | 0.105 |  | 0.050 | 0.040 | 0.050 | 0.052 | 0.060 | 0.126 |
|  | (with $X_{r}$) | 10% | -0.101 | -0.017 | 0.006 | 0.022 | 0.069 | 0.187 |  | -0.096 | -0.022 | 0.011 | 0.030 | 0.077 | 0.187 |
|  |  | 5% | -1.087 | -0.502 | -0.218 | -0.051 | 0.447 | 1.593 |  | -0.989 | -0.430 | -0.203 | -0.056 | 0.447 | 1.750 |
| MSE | $\pi$ | 50% | 0.229 | 0.229 | 0.245 | 0.260 | 0.336 | 0.865 |  | 0.144 | 0.145 | 0.151 | 0.160 | 0.202 | 0.862 |
|  |  | 10% | 1.835 | 0.702 | 0.555 | 0.495 | 0.611 | 1.618 |  | 1.686 | 0.729 | 0.459 | 0.342 | 0.326 | 1.431 |
|  |  | 5% | 22.016 | 11.761 | 7.358 | 5.613 | 7.516 | 25.242 |  | 19.574 | 9.400 | 6.274 | 4.973 | 7.605 | 27.879 |
|  | $\pi^{*}$ | 50% | 0.187 | 0.190 | 0.202 | 0.214 | 0.271 | 0.711 |  | 0.174 | 0.182 | 0.193 | 0.206 | 0.261 | 0.880 |
|  |  | 10% | 1.636 | 0.616 | 0.493 | 0.448 | 0.605 | 1.702 |  | 1.640 | 0.774 | 0.532 | 0.424 | 0.421 | 1.584 |
|  |  | 5% | 20.447 | 10.945 | 6.941 | 5.395 | 8.031 | 27.171 |  | 18.505 | 9.022 | 6.120 | 5.002 | 8.210 | 29.796 |
| Coverage | $\pi$ | 50% | 93.3 | 93.6 | 93.2 | 92.9 | 92.5 | 90.6 |  | 92.4 | 93.5 | 94.0 | 94.1 | 95.2 | 95.8 |
| (%) |  | 10% | 91.8 | 92.5 | 92.5 | 93.0 | 93.2 | 93.9 |  | 93.9 | 94.2 | 94.2 | 94.6 | 95.1 | 96.6 |
|  |  | 5% | 88.4 | 90.3 | 91.2 | 91.7 | 92.1 | 85.8 |  | 89.8 | 93.1 | 94.6 | 95.5 | 95.5 | 88.4 |
|  | $\pi^{*}$ | 50% | 94.2 | 94.4 | 94.4 | 93.8 | 93.8 | 93.4 |  | 94.5 | 94.2 | 94.0 | 94.3 | 93.6 | 94.0 |
|  |  | 10% | 93.6 | 93.7 | 93.2 | 93.6 | 93.2 | 93.6 |  | 94.0 | 94.0 | 94.0 | 93.9 | 93.9 | 93.7 |
|  |  | 5% | 89.3 | 92.0 | 92.8 | 92.9 | 92.9 | 87.2 |  | 90.0 | 92.9 | 93.4 | 93.6 | 93.4 | 86.8 |
| Type I error | $\pi$ | 50% | 6.5 | 16.7 | 35.7 | 52.0 | 88.0 | 96.7 |  | 7.5 | 35.9 | 68.6 | 86.2 | 99.9 | 100.0 |
| and power |  | 10% | 8.1 | 11.5 | 21.5 | 33.5 | 74.9 | 94.7 |  | 6.0 | 20.3 | 39.1 | 56.2 | 93.1 | 99.5 |
| (%) |  | 5% | 11.4 | 11.6 | 16.7 | 23.4 | 51.9 | 78.2 |  | 10.2 | 15.1 | 25.2 | 35.6 | 72.3 | 92.3 |
|  | $\pi^{*}$ | 50% | 5.6 | 19.6 | 41.2 | 59.9 | 92.8 | 98.1 |  | 5.4 | 20.1 | 44.4 | 63.4 | 94.1 | 98.7 |
|  |  | 10% | 6.1 | 13.5 | 26.4 | 40.2 | 79.3 | 95.5 |  | 5.9 | 13.5 | 26.6 | 40.8 | 81.1 | 96.2 |
|  |  | 5% | 10.6 | 13.7 | 21.0 | 29.2 | 60.3 | 83.1 |  | 9.9 | 13.2 | 20.7 | 29.0 | 60.8 | 84.4 |

[Note] Scenario I: the situation wherein the covariate distribution is similar between the randomized control trial (RCT) data and historical control data; Scenario II: the situation wherein the covariate distribution is not similar between the RCT data and historical control data; $\pi$ (without $X_{r}$): the conventional method; $\pi^{*}$ (with $X_{r}$): the proposed method.

1. **Simulation setting assuming that the allocation ratio between the RCT treatment group, RCT control group, and historical control data is other than 1:1:2**

In the text, the simulation results assuming an allocation ratio between the RCT treatment group, RCT control group, and historical control group as 1:1:2 are presented. However, other allocation ratios were also examined. When the allocation ratios between the RCT and historical control data are 1:1, 1:2, and 1:9, the cases wherein the ratios between the RCT treatment group and RCT control group were 1:1, 2:1, and 9:1 were examined, respectively (Appendix Table B.1). For example, the allocation ratio in the text is the case wherein the ratio between RCT data historical control data is 1:1 and that between the RCT treatment group and RCT control group is 1:1.


Allocation ratios between the simulated randomized control trial (RCT) treatment group, RCT control group, and historical control data

| **Rt:Rc:H** | **t:c = 1:1** | **t:c = 2:1** | **t:c = 9:1** |
| --- | --- | --- | --- |
| **R:H = 1:1** | 1：1：2 | 2：1：3 | 9：1：10 |
| **R:H = 1:2** | 1：1：4 | 2：1：6 | 9：1：20 |
| **R:H = 1:9** | 1：1：18 | 2：1：27 | 9：1：90 |

[Note] R: RCT, H：Historical control, t: treatment group, c: control group.

Moreover, when the allocation ratios between the RCT treatment group and RCT control + historical control data are 1:1—that is, the allocation ratios between the overall treatment and overall control are 1:1—the cases wherein ratios between the RCT control group and historical control data were 1:1 and 1:2 are examined (Appendix Table B.2).


Allocation ratios between the simulated randomized control trial (RCT) treatment group, RCT control group, and historical control data

| **Rt:Rc:H** | **Rc:H = 1:1** | **Rc:H = 1:2** |
| --- | --- | --- |
| **Rt:(Rc+H) = 1:1** | 2：1：1 | 3：1：2 |

[Note] R: RCT, H：Historical control, t: treatment group, c: control group.

The simulation results assuming allocation ratios other than those dealt with in the main text are shown below. The method and conditions in the simulation setting are the same as those shown in the text, except for the allocation ratio.


Performance of the estimated propensity score (PS) model by simulation setting assuming Rt:Rc:H = 2:1:3

|  |  |  | **Scenario Ⅰ** | | | | | |  | **Scenario Ⅱ** | | | | | |
| --- | --- | --- | --- | --- | --- | --- | --- | --- | --- | --- | --- | --- | --- | --- | --- |
|  |  |  | Odds ratio | | | | | |  | Odds ratio | | | | | |
| Performance measurement | PS model | Outcome event rate | 1.0 | 1.5 | 2.0 | 2.5 | 5.0 | 10.0 |  | 1.0 | 1.5 | 2.0 | 2.5 | 5.0 | 10.0 |
| Bias | $\pi$ | 50% | 0.002 | -0.010 | -0.018 | -0.023 | -0.035 | -0.045 |  | 0.180 | 0.168 | 0.161 | 0.155 | 0.142 | 0.132 |
|  | (without $X_{r}$) | 10% | -0.015 | -0.012 | -0.012 | -0.012 | -0.004 | 0.004 |  | 0.167 | 0.170 | 0.169 | 0.169 | 0.168 | 0.178 |
|  |  | 5% | -0.031 | -0.016 | -0.006 | 0.002 | 0.018 | 0.075 |  | 0.156 | 0.164 | 0.170 | 0.175 | 0.194 | 0.232 |
|  | $\pi^{*}$ | 50% | 0.043 | 0.032 | 0.023 | 0.016 | 0.003 | -0.008 |  | 0.043 | 0.033 | 0.026 | 0.020 | 0.007 | -0.004 |
|  | (with $X_{r}$) | 10% | 0.046 | 0.047 | 0.048 | 0.049 | 0.062 | 0.079 |  | 0.040 | 0.046 | 0.046 | 0.049 | 0.056 | 0.077 |
|  |  | 5% | 0.050 | 0.062 | 0.073 | 0.081 | 0.109 | 0.181 |  | 0.037 | 0.051 | 0.059 | 0.069 | 0.102 | 0.166 |
| MSE | $\pi$ | 50% | 0.033 | 0.034 | 0.035 | 0.037 | 0.044 | 0.058 |  | 0.053 | 0.049 | 0.048 | 0.047 | 0.047 | 0.053 |
|  |  | 10% | 0.089 | 0.081 | 0.079 | 0.079 | 0.088 | 0.117 |  | 0.086 | 0.083 | 0.082 | 0.083 | 0.093 | 0.122 |
|  |  | 5% | 0.169 | 0.156 | 0.152 | 0.152 | 0.179 | 0.447 |  | 0.143 | 0.135 | 0.136 | 0.139 | 0.170 | 0.340 |
|  | $\pi^{*}$ | 50% | 0.032 | 0.033 | 0.032 | 0.034 | 0.039 | 0.049 |  | 0.032 | 0.031 | 0.032 | 0.034 | 0.039 | 0.049 |
|  |  | 10% | 0.090 | 0.088 | 0.090 | 0.094 | 0.117 | 0.168 |  | 0.088 | 0.087 | 0.089 | 0.092 | 0.115 | 0.165 |
|  |  | 5% | 0.170 | 0.170 | 0.175 | 0.184 | 0.241 | 0.582 |  | 0.169 | 0.164 | 0.169 | 0.176 | 0.233 | 0.455 |
| Coverage | $\pi$ | 50% | 95.3 | 95.1 | 94.9 | 94.8 | 94.8 | 94.2 |  | 76.7 | 80.2 | 82.1 | 84.0 | 87.6 | 90.4 |
| (%) |  | 10% | 94.2 | 94.4 | 94.6 | 94.5 | 94.4 | 94.7 |  | 88.8 | 88.7 | 89.0 | 89.2 | 90.6 | 93.1 |
|  |  | 5% | 94.0 | 93.8 | 94.2 | 94.5 | 94.4 | 94.8 |  | 91.6 | 91.6 | 91.7 | 92.2 | 94.0 | 95.9 |
|  | $\pi^{*}$ | 50% | 94.5 | 94.8 | 95.6 | 95.0 | 95.3 | 94.6 |  | 94.7 | 95.1 | 95.1 | 94.8 | 95.0 | 94.8 |
|  |  | 10% | 93.7 | 93.6 | 93.3 | 93.0 | 92.5 | 91.6 |  | 93.9 | 93.4 | 93.3 | 93.0 | 92.3 | 91.5 |
|  |  | 5% | 93.1 | 93.0 | 92.3 | 92.2 | 91.3 | 91.0 |  | 93.2 | 93.1 | 92.7 | 92.5 | 91.6 | 91.4 |
| Type I error | $\pi$ | 50% | 4.7 | 57.8 | 94.4 | 99.3 | 100.0 | 100.0 |  | 23.2 | 97.6 | 100.0 | 100.0 | 100.0 | 100.0 |
| and power |  | 10% | 5.7 | 30.3 | 70.2 | 91.1 | 99.9 | 100.0 |  | 11.2 | 69.5 | 95.8 | 99.4 | 100.0 | 100.0 |
| (%) |  | 5% | 6.0 | 20.4 | 47.1 | 70.1 | 98.6 | 99.9 |  | 8.4 | 44.0 | 76.6 | 92.0 | 100.0 | 100.0 |
|  | $\pi^{*}$ | 50% | 5.5 | 69.0 | 97.6 | 99.8 | 100.0 | 100.0 |  | 5.3 | 70.6 | 97.8 | 99.8 | 100.0 | 100.0 |
|  |  | 10% | 6.4 | 38.6 | 73.2 | 89.1 | 99.2 | 99.8 |  | 6.1 | 38.7 | 72.7 | 89.2 | 99.2 | 99.8 |
|  |  | 5% | 6.9 | 27.5 | 54.0 | 72.0 | 95.4 | 98.9 |  | 6.7 | 26.9 | 52.5 | 71.6 | 95.3 | 98.9 |

[Note] Scenario I: the situation wherein the covariate distribution is similar between the randomized control trial (RCT) data and historical control data; Scenario II: the situation wherein the covariate distribution is not similar between the RCT data and historical control data; $\pi$ (without $X_{r}$): the conventional method; $\pi^{*}$ (with $X_{r}$): the proposed method.

Performance of the estimated propensity score (PS) model by simulation setting assuming Rt:Rc:H = 9:1:10

|  |  |  | **Scenario Ⅰ** | | | | | |  | **Scenario Ⅱ** | | | | | |
| --- | --- | --- | --- | --- | --- | --- | --- | --- | --- | --- | --- | --- | --- | --- | --- |
|  |  |  | Odds ratio | | | | | |  | Odds ratio | | | | | |
| Performance measurement | PS model | Outcome event rate | 1.0 | 1.5 | 2.0 | 2.5 | 5.0 | 10.0 |  | 1.0 | 1.5 | 2.0 | 2.5 | 5.0 | 10.0 |
| Bias | $\pi$ | 50% | 0.000 | -0.012 | -0.021 | -0.027 | -0.042 | -0.056 |  | 0.217 | 0.207 | 0.199 | 0.192 | 0.175 | 0.161 |
|  | (without $X_{r}$) | 10% | -0.002 | -0.002 | -0.003 | 0.000 | 0.011 | 0.037 |  | 0.218 | <-10^9^ | <-10^9^ | <-10^9^ | <-10^9^ | <-10^9^ |
|  |  | 5% | -0.008 | 0.005 | 0.012 | 0.023 | 0.064 | 0.273 |  | <-10^9^ | <-10^9^ | <-10^9^ | <-10^9^ | 0.294 | 0.475 |
|  | $\pi^{*}$ | 50% | 0.054 | 0.045 | 0.037 | 0.033 | 0.024 | 0.017 |  | 0.045 | 0.037 | 0.032 | 0.028 | 0.020 | 0.009 |
|  | (with $X_{r}$) | 10% | 0.115 | 0.123 | 0.133 | 0.148 | 0.192 | 0.269 |  | 0.108 | 0.123 | 0.136 | 0.145 | 0.187 | 0.266 |
|  |  | 5% | 0.164 | 0.190 | 0.207 | 0.229 | 0.321 | 0.600 |  | 0.156 | 0.181 | 0.205 | 0.226 | 0.310 | 0.544 |
| MSE | $\pi$ | 50% | 0.026 | 0.027 | 0.028 | 0.029 | 0.033 | 0.041 |  | 0.075 | 0.070 | 0.068 | 0.066 | 0.064 | 0.065 |
|  |  | 10% | 0.072 | 0.074 | 0.077 | 0.082 | 0.110 | 0.179 |  | 0.127 | >10^24^ | >10^24^ | >10^24^ | >10^24^ | >10^24^ |
|  |  | 5% | 0.142 | 0.144 | 0.153 | 0.167 | 0.304 | 2.925 |  | >10^24^ | >10^24^ | >10^24^ | >10^24^ | 0.368 | 2.675 |
|  | $\pi^{*}$ | 50% | 0.075 | 0.076 | 0.078 | 0.081 | 0.088 | 0.098 |  | 0.077 | 0.078 | 0.081 | 0.081 | 0.089 | 0.101 |
|  |  | 10% | 0.204 | 0.225 | 0.248 | 0.273 | 0.372 | 0.544 |  | 0.209 | 0.227 | 0.249 | 0.270 | 0.371 | 0.537 |
|  |  | 5% | 0.349 | 0.390 | 0.428 | 0.466 | 0.726 | 3.792 |  | 0.348 | 0.389 | 0.430 | 0.470 | 0.675 | 3.294 |
| Coverage | $\pi$ | 50% | 95.3 | 95.3 | 95.0 | 95.1 | 94.8 | 94.5 |  | 71.5 | 74.1 | 76.2 | 77.5 | 82.0 | 85.3 |
| (%) |  | 10% | 95.0 | 95.3 | 94.8 | 94.8 | 94.6 | 94.4 |  | 84.5 | 84.4 | 84.7 | 85.3 | 86.3 | 88.9 |
|  |  | 5% | 94.5 | 94.5 | 94.7 | 94.4 | 94.6 | 94.8 |  | 87.7 | 87.7 | 88.1 | 88.0 | 90.3 | 93.5 |
|  | $\pi^{*}$ | 50% | 93.4 | 93.2 | 93.5 | 93.1 | 92.7 | 92.6 |  | 93.2 | 92.9 | 92.9 | 92.5 | 92.8 | 92.1 |
|  |  | 10% | 85.7 | 84.2 | 82.6 | 81.1 | 76.4 | 72.6 |  | 85.8 | 84.0 | 82.6 | 81.2 | 75.9 | 72.6 |
|  |  | 5% | 82.2 | 79.5 | 77.9 | 76.8 | 73.9 | 80.4 |  | 82.8 | 79.6 | 77.8 | 76.5 | 75.3 | 81.8 |
| Type I error | $\pi$ | 50% | 4.7 | 66.3 | 98.1 | 99.9 | 100.0 | 100.0 |  | 28.5 | 95.5 | 99.3 | 99.6 | 99.9 | 100.0 |
| and power |  | 10% | 5.0 | 32.9 | 71.2 | 90.1 | 99.7 | 99.9 |  | 15.4 | 67.2 | 89.9 | 95.7 | 99.1 | 99.7 |
| (%) |  | 5% | 5.5 | 20.6 | 47.4 | 68.3 | 96.1 | 99.1 |  | 12.2 | 46.0 | 72.4 | 85.2 | 97.4 | 99.0 |
|  | $\pi^{*}$ | 50% | 6.6 | 46.3 | 79.9 | 92.2 | 99.2 | 99.9 |  | 6.8 | 44.3 | 78.3 | 91.7 | 99.3 | 99.8 |
|  |  | 10% | 14.3 | 39.2 | 57.9 | 69.7 | 88.7 | 95.1 |  | 14.2 | 39.4 | 58.3 | 69.1 | 88.4 | 94.9 |
|  |  | 5% | 17.7 | 39.5 | 54.4 | 63.9 | 82.4 | 91.0 |  | 17.2 | 39.9 | 54.8 | 64.3 | 82.6 | 91.1 |

[Note] Scenario I: the situation wherein the covariate distribution is similar between the randomized control trial (RCT) data and historical control data; Scenario II: the situation wherein the covariate distribution is not similar between the RCT data and historical control data; $\pi$ (without $X_{r}$): the conventional method; $\pi^{*}$ (with $X_{r}$): the proposed method.


Performance of the estimated propensity score (PS) model by simulation setting assuming Rt:Rc:Hc = 1:1:4

|  |  |  | **Scenario Ⅰ** | | | | | |  | **Scenario Ⅱ** | | | | | |
| --- | --- | --- | --- | --- | --- | --- | --- | --- | --- | --- | --- | --- | --- | --- | --- |
|  |  |  | Odds ratio | | | | | |  | Odds ratio | | | | | |
| Performance measurement | PS model | Outcome event rate | 1.0 | 1.5 | 2.0 | 2.5 | 5.0 | 10.0 |  | 1.0 | 1.5 | 2.0 | 2.5 | 5.0 | 10.0 |
| Bias | $\pi$ | 50% | 0.003 | -0.002 | -0.006 | -0.009 | -0.009 | <-10^9^ |  | 0.200 | 0.189 | 0.182 | 0.177 | 0.169 | 0.173 |
|  | (without $X_{r}$) | 10% | -0.062 | -0.048 | -0.040 | -0.037 | -0.034 | -0.033 |  | 0.169 | 0.173 | 0.174 | 0.172 | 0.168 | 0.163 |
|  |  | 5% | -0.128 | -0.087 | -0.069 | -0.061 | -0.040 | -0.020 |  | 0.130 | 0.154 | 0.163 | 0.171 | 0.182 | 0.191 |
|  | $\pi^{*}$ | 50% | 0.046 | 0.038 | 0.033 | 0.028 | 0.022 | 0.031 |  | 0.060 | 0.049 | 0.045 | 0.040 | 0.035 | 0.046 |
|  | (with $X_{r}$) | 10% | 0.007 | 0.015 | 0.019 | 0.020 | 0.019 | 0.020 |  | 0.030 | 0.036 | 0.039 | 0.038 | 0.036 | 0.034 |
|  |  | 5% | -0.030 | -0.003 | 0.009 | 0.013 | 0.029 | 0.047 |  | -0.016 | 0.014 | 0.025 | 0.035 | 0.052 | 0.067 |
| MSE | $\pi$ | 50% | 0.075 | 0.078 | 0.083 | 0.089 | 0.120 | >10^24^ |  | 0.075 | 0.072 | 0.071 | 0.072 | 0.084 | 0.117 |
|  |  | 10% | 0.196 | 0.156 | 0.136 | 0.126 | 0.108 | 0.104 |  | 0.124 | 0.108 | 0.100 | 0.096 | 0.089 | 0.089 |
|  |  | 5% | 0.514 | 0.292 | 0.250 | 0.227 | 0.187 | 0.182 |  | 0.290 | 0.184 | 0.162 | 0.153 | 0.143 | 0.158 |
|  | $\pi^{*}$ | 50% | 0.054 | 0.055 | 0.058 | 0.061 | 0.083 | 0.126 |  | 0.050 | 0.052 | 0.056 | 0.058 | 0.078 | 0.119 |
|  |  | 10% | 0.135 | 0.111 | 0.100 | 0.094 | 0.085 | 0.089 |  | 0.127 | 0.105 | 0.095 | 0.090 | 0.084 | 0.088 |
|  |  | 5% | 0.374 | 0.207 | 0.182 | 0.169 | 0.153 | 0.167 |  | 0.313 | 0.205 | 0.177 | 0.164 | 0.151 | 0.166 |
| Coverage | $\pi$ | 50% | 94.1 | 94.6 | 93.8 | 93.6 | 93.2 | 92.1 |  | 81.7 | 83.9 | 85.9 | 87.5 | 91.2 | 93.1 |
| (%) |  | 10% | 92.0 | 92.7 | 93.4 | 93.6 | 94.1 | 94.7 |  | 89.5 | 89.0 | 88.7 | 88.5 | 89.1 | 90.6 |
|  |  | 5% | 91.0 | 91.5 | 92.0 | 92.3 | 93.4 | 94.1 |  | 91.6 | 91.0 | 91.1 | 90.9 | 91.4 | 92.8 |
|  | $\pi^{*}$ | 50% | 94.2 | 94.4 | 94.3 | 94.5 | 94.3 | 93.6 |  | 94.2 | 94.0 | 93.9 | 94.3 | 94.4 | 94.1 |
|  |  | 10% | 94.0 | 94.1 | 94.4 | 94.5 | 94.6 | 94.9 |  | 93.9 | 93.9 | 94.4 | 94.5 | 94.0 | 94.4 |
|  |  | 5% | 94.0 | 93.9 | 94.0 | 94.3 | 94.2 | 94.6 |  | 93.7 | 93.7 | 93.9 | 93.8 | 93.6 | 93.9 |
| Type I error | $\pi$ | 50% | 5.9 | 35.0 | 70.1 | 87.5 | 98.9 | 99.7 |  | 18.2 | 88.4 | 99.6 | 100.0 | 100.0 | 100.0 |
| and power |  | 10% | 8.0 | 19.8 | 49.3 | 74.8 | 99.6 | 100.0 |  | 10.5 | 56.2 | 89.1 | 98.0 | 100.0 | 100.0 |
| (%) |  | 5% | 9.0 | 14.4 | 33.4 | 54.2 | 96.3 | 99.9 |  | 8.4 | 36.6 | 67.2 | 85.9 | 99.9 | 100.0 |
|  | $\pi^{*}$ | 50% | 5.8 | 51.1 | 87.1 | 96.4 | 99.8 | 99.9 |  | 5.7 | 55.2 | 90.0 | 98.1 | 99.9 | 100.0 |
|  |  | 10% | 5.9 | 29.2 | 64.9 | 87.4 | 99.9 | 100.0 |  | 6.1 | 32.9 | 69.2 | 89.1 | 100.0 | 100.0 |
|  |  | 5% | 6.0 | 20.1 | 45.3 | 67.4 | 98.0 | 99.9 |  | 6.2 | 22.3 | 48.3 | 70.4 | 98.7 | 100.0 |

[Note] Scenario I: the situation wherein the covariate distribution is similar between the randomized control trial (RCT) data and historical control data; Scenario II: the situation wherein the covariate distribution is not similar between the RCT data and historical control data; $\pi$ (without $X_{r}$): the conventional method; $\pi^{*}$ (with $X_{r}$): the proposed method.


Performance of the estimated propensity score (PS) model by simulation setting assuming Rt:Rc:Hc = 2:1:6

|  |  |  | **Scenario Ⅰ** | | | | | |  | **Scenario Ⅱ** | | | | | |
| --- | --- | --- | --- | --- | --- | --- | --- | --- | --- | --- | --- | --- | --- | --- | --- |
|  |  |  | Odds ratio | | | | | |  | Odds ratio | | | | | |
| Performance measurement | PS model | Outcome event rate | 1.0 | 1.5 | 2.0 | 2.5 | 5.0 | 10.0 |  | 1.0 | 1.5 | 2.0 | 2.5 | 5.0 | 10.0 |
| Bias | $\pi$ | 50% | 0.002 | -0.005 | -0.011 | -0.016 | -0.025 | -0.020 |  | 0.207 | 0.195 | 0.187 | 0.183 | 0.172 | 0.171 |
|  | (without $X_{r}$) | 10% | -0.041 | -0.031 | -0.030 | -0.029 | -0.027 | -0.024 |  | 0.182 | 0.187 | 0.186 | 0.185 | 0.182 | 0.181 |
|  |  | 5% | -0.087 | -0.058 | -0.046 | -0.039 | -0.019 | 0.002 |  | 0.155 | 0.172 | 0.178 | 0.186 | 0.199 | 0.214 |
|  | $\pi^{*}$ | 50% | 0.058 | 0.048 | 0.041 | 0.036 | 0.023 | 0.020 |  | 0.058 | 0.048 | 0.041 | 0.036 | 0.025 | 0.026 |
|  | (with $X_{r}$) | 10% | 0.043 | 0.049 | 0.049 | 0.049 | 0.049 | 0.054 |  | 0.046 | 0.052 | 0.052 | 0.053 | 0.054 | 0.055 |
|  |  | 5% | 0.024 | 0.044 | 0.052 | 0.058 | 0.077 | 0.104 |  | 0.025 | 0.044 | 0.052 | 0.061 | 0.082 | 0.108 |
| MSE | $\pi$ | 50% | 0.053 | 0.054 | 0.057 | 0.061 | 0.080 | 0.117 |  | 0.072 | 0.067 | 0.066 | 0.066 | 0.071 | 0.092 |
|  |  | 10% | 0.138 | 0.116 | 0.105 | 0.100 | 0.091 | 0.098 |  | 0.114 | 0.103 | 0.097 | 0.095 | 0.093 | 0.101 |
|  |  | 5% | 0.274 | 0.213 | 0.190 | 0.180 | 0.167 | 0.189 |  | 0.225 | 0.167 | 0.155 | 0.153 | 0.155 | 0.192 |
|  | $\pi^{*}$ | 50% | 0.041 | 0.041 | 0.041 | 0.043 | 0.052 | 0.074 |  | 0.039 | 0.038 | 0.039 | 0.041 | 0.051 | 0.073 |
|  |  | 10% | 0.103 | 0.093 | 0.089 | 0.088 | 0.091 | 0.111 |  | 0.101 | 0.090 | 0.087 | 0.085 | 0.090 | 0.107 |
|  |  | 5% | 0.204 | 0.173 | 0.164 | 0.163 | 0.172 | 0.222 |  | 0.224 | 0.170 | 0.162 | 0.159 | 0.171 | 0.224 |
| Coverage | $\pi$ | 50% | 94.2 | 94.5 | 94.5 | 94.3 | 93.9 | 93.0 |  | 77.3 | 80.5 | 82.6 | 84.3 | 88.2 | 91.1 |
| (%) |  | 10% | 92.9 | 93.4 | 93.6 | 94.0 | 94.3 | 94.1 |  | 88.0 | 87.4 | 87.9 | 88.0 | 88.9 | 90.3 |
|  |  | 5% | 92.1 | 92.7 | 92.9 | 93.0 | 93.7 | 94.5 |  | 90.7 | 90.5 | 90.4 | 90.5 | 91.3 | 93.2 |
|  | $\pi^{*}$ | 50% | 93.6 | 94.2 | 94.7 | 94.9 | 95.2 | 94.8 |  | 94.0 | 94.7 | 95.2 | 94.8 | 95.0 | 94.5 |
|  |  | 10% | 93.9 | 93.5 | 93.6 | 93.4 | 93.2 | 92.8 |  | 93.5 | 93.7 | 93.4 | 93.3 | 93.6 | 93.0 |
|  |  | 5% | 93.5 | 93.6 | 93.3 | 93.1 | 93.0 | 92.6 |  | 93.6 | 93.3 | 93.0 | 93.0 | 92.3 | 92.3 |
| Type I error | $\pi$ | 50% | 5.7 | 43.8 | 82.4 | 94.9 | 99.7 | 99.9 |  | 22.6 | 93.9 | 99.9 | 100.0 | 100.0 | 100.0 |
| and power |  | 10% | 7.1 | 23.5 | 58.8 | 83.6 | 99.9 | 100.0 |  | 12.0 | 62.5 | 92.4 | 99.0 | 100.0 | 100.0 |
| (%) |  | 5% | 7.9 | 15.5 | 38.6 | 61.6 | 98.3 | 100.0 |  | 9.2 | 39.6 | 71.6 | 89.1 | 100.0 | 100.0 |
|  | $\pi^{*}$ | 50% | 6.4 | 65.0 | 95.5 | 99.4 | 100.0 | 100.0 |  | 6.0 | 66.9 | 96.4 | 99.6 | 100.0 | 100.0 |
|  |  | 10% | 6.1 | 38.0 | 73.1 | 90.1 | 99.6 | 99.9 |  | 6.5 | 38.5 | 74.5 | 91.0 | 99.6 | 99.9 |
|  |  | 5% | 6.5 | 26.1 | 53.1 | 72.9 | 97.3 | 99.5 |  | 6.4 | 26.7 | 53.3 | 73.4 | 97.5 | 99.5 |

[Note] Scenario I: the situation wherein the covariate distribution is similar between the randomized control trial (RCT) data and historical control data; Scenario II: the situation wherein the covariate distribution is not similar between the RCT data and historical control data; $\pi$ (without $X_{r}$): the conventional method; $\pi^{*}$ (with $X_{r}$): the proposed method.


Performance of the estimated propensity score (PS) model by simulation setting assuming Rt:Rc:Hc = 9:1:20

|  |  |  | **Scenario Ⅰ** | | | | | |  | **Scenario Ⅱ** | | | | | |
| --- | --- | --- | --- | --- | --- | --- | --- | --- | --- | --- | --- | --- | --- | --- | --- |
|  |  |  | Odds ratio | | | | | |  | Odds ratio | | | | | |
| Performance measurement | PS model | Outcome event rate | 1.0 | 1.5 | 2.0 | 2.5 | 5.0 | 10.0 |  | 1.0 | 1.5 | 2.0 | 2.5 | 5.0 | 10.0 |
| Bias | $\pi$ | 50% | -0.003 | -0.014 | -0.021 | -0.024 | -0.038 | -0.042 |  | 0.240 | 0.229 | 0.221 | 0.215 | 0.202 | 0.194 |
|  | (without $X_{r}$) | 10% | -0.017 | -0.015 | -0.014 | -0.014 | -0.012 | -0.003 |  | 0.222 | 0.226 | 0.226 | 0.226 | 0.227 | 0.236 |
|  |  | 5% | -0.043 | -0.023 | -0.016 | -0.010 | 0.014 | 0.046 |  | 0.205 | 0.219 | 0.226 | 0.232 | 0.254 | 0.285 |
|  | $\pi^{*}$ | 50% | 0.073 | 0.064 | 0.058 | 0.052 | 0.036 | 0.031 |  | 0.060 | 0.051 | 0.043 | 0.040 | 0.030 | 0.022 |
|  | (with $X_{r}$) | 10% | 0.110 | 0.113 | 0.117 | 0.119 | 0.138 | <-10^9^ |  | 0.094 | 0.103 | 0.106 | 0.109 | 0.124 | 0.157 |
|  |  | 5% | 0.130 | <-10^9^ | <-10^9^ | <-10^9^ | <-10^9^ | <-10^9^ |  | 0.114 | 0.132 | 0.143 | 0.155 | 0.196 | 0.250 |
| MSE | $\pi$ | 50% | 0.038 | 0.039 | 0.040 | 0.043 | 0.053 | 0.072 |  | 0.089 | 0.084 | 0.081 | 0.081 | 0.081 | 0.091 |
|  |  | 10% | 0.097 | 0.087 | 0.083 | 0.082 | 0.086 | 0.105 |  | 0.138 | 0.131 | 0.128 | 0.127 | 0.136 | 0.163 |
|  |  | 5% | 0.187 | 0.163 | 0.154 | 0.153 | 0.169 | 0.283 |  | 0.219 | 0.205 | 0.202 | 0.204 | 0.230 | 0.312 |
|  | $\pi^{*}$ | 50% | 0.068 | 0.067 | 0.068 | 0.068 | 0.074 | 0.084 |  | 0.064 | 0.064 | 0.065 | 0.067 | 0.072 | 0.082 |
|  |  | 10% | 0.168 | 0.171 | 0.176 | 0.183 | 0.220 | >10^24^ |  | 0.165 | 0.165 | 0.170 | 0.177 | 0.212 | 0.270 |
|  |  | 5% | 0.275 | >10^24^ | >10^24^ | >10^24^ | >10^24^ | >10^24^ |  | 0.273 | 0.275 | 0.285 | 0.294 | 0.351 | 0.471 |
| Coverage | $\pi$ | 50% | 94.7 | 94.4 | 94.8 | 94.5 | 94.0 | 93.7 |  | 71.7 | 74.6 | 76.6 | 78.0 | 82.3 | 86.9 |
| (%) |  | 10% | 94.3 | 93.9 | 94.3 | 94.3 | 94.7 | 94.4 |  | 85.8 | 85.0 | 85.2 | 85.5 | 86.6 | 88.0 |
|  |  | 5% | 93.6 | 94.1 | 94.1 | 94.1 | 94.1 | 94.5 |  | 89.0 | 88.5 | 88.3 | 88.4 | 89.4 | 91.8 |
|  | $\pi^{*}$ | 50% | 92.7 | 93.0 | 92.8 | 93.2 | 93.4 | 93.9 |  | 93.4 | 93.2 | 93.6 | 93.1 | 93.6 | 94.0 |
|  |  | 10% | 87.0 | 85.6 | 84.5 | 84.1 | 81.6 | 80.7 |  | 87.5 | 86.6 | 85.9 | 84.8 | 83.0 | 81.9 |
|  |  | 5% | 86.7 | 84.8 | 84.0 | 82.7 | 82.1 | 85.1 |  | 87.1 | 85.4 | 84.5 | 84.1 | 83.9 | 86.4 |
| Type I error | $\pi$ | 50% | 5.3 | 52.5 | 91.9 | 98.8 | 100.0 | 100.0 |  | 28.2 | 94.2 | 99.6 | 99.9 | 100.0 | 100.0 |
| and power |  | 10% | 5.6 | 28.8 | 68.0 | 89.8 | 100.0 | 100.0 |  | 14.2 | 63.8 | 91.3 | 97.7 | 99.6 | 99.9 |
| (%) |  | 5% | 6.4 | 18.4 | 46.0 | 68.6 | 98.6 | 100.0 |  | 11.0 | 42.7 | 71.2 | 87.2 | 99.0 | 99.7 |
|  | $\pi^{*}$ | 50% | 7.3 | 55.4 | 86.9 | 95.5 | 99.7 | 99.9 |  | 6.6 | 53.7 | 86.1 | 95.1 | 99.7 | 99.9 |
|  |  | 10% | 13.0 | 44.7 | 67.3 | 78.8 | 93.5 | 97.7 |  | 12.4 | 44.9 | 67.0 | 78.8 | 93.9 | 97.7 |
|  |  | 5% | 13.2 | 40.6 | 61.4 | 73.3 | 89.9 | 95.6 |  | 12.8 | 39.6 | 60.1 | 71.7 | 89.4 | 95.1 |

[Note] Scenario I: the situation wherein the covariate distribution is similar between the randomized control trial (RCT) data and historical control data; Scenario II: the situation wherein the covariate distribution is not similar between the RCT data and historical control data; $\pi$ (without $X_{r}$): the conventional method; $\pi^{*}$ (with $X_{r}$): the proposed method.


Performance of the estimated propensity score (PS) model by simulation setting assuming Rt:Rc:Hc = 1:1:18

|  |  |  | **Scenario Ⅰ** | | | | | |  | **Scenario Ⅱ** | | | | | |
| --- | --- | --- | --- | --- | --- | --- | --- | --- | --- | --- | --- | --- | --- | --- | --- |
|  |  |  | Odds ratio | | | | | |  | Odds ratio | | | | | |
| Performance measurement | PS model | Outcome event rate | 1.0 | 1.5 | 2.0 | 2.5 | 5.0 | 10.0 |  | 1.0 | 1.5 | 2.0 | 2.5 | 5.0 | 10.0 |
| Bias | $\pi$ | 50% | 0.013 | 0.029 | >10^9^ | >10^9^ | >10^9^ | >10^9^ |  | 0.246 | 0.247 | 0.255 | 0.261 | 0.300 | 0.620 |
|  | (without $X_{r}$) | 10% | <-10^9^ | <-10^9^ | <-10^9^ | <-10^9^ | <-10^9^ | -0.045 |  | <-10^9^ | <-10^9^ | <-10^9^ | <-10^9^ | 0.175 | 0.192 |
|  |  | 5% | <-10^9^ | <-10^9^ | <-10^9^ | <-10^9^ | <-10^9^ | <-10^9^ |  | -1.218 | <-10^9^ | <-10^9^ | <-10^9^ | <-10^9^ | 0.177 |
|  | $\pi^{*}$ | 50% | 0.070 | 0.076 | 0.079 | 0.086 | <-10^9^ | <-10^9^ |  | 0.080 | 0.086 | 0.095 | 0.099 | 0.144 | 0.442 |
|  | (with $X_{r}$) | 10% | -0.161 | -0.042 | -0.014 | -0.006 | 0.017 | 0.020 |  | -0.137 | -0.026 | -0.003 | 0.008 | 0.025 | 0.028 |
|  |  | 5% | -1.304 | -0.524 | -0.227 | -0.118 | -0.008 | 0.015 |  | -1.183 | -0.458 | -0.207 | -0.101 | 0.000 | 0.026 |
| MSE | $\pi$ | 50% | 0.312 | 0.339 | >10^24^ | >10^24^ | >10^24^ | >10^24^ |  | 0.196 | 0.205 | 0.222 | 0.245 | 0.578 | 5.706 |
|  |  | 10% | >10^24^ | >10^24^ | >10^24^ | >10^24^ | >10^24^ | 0.323 |  | >10^24^ | >10^24^ | >10^24^ | >10^24^ | 0.189 | 0.186 |
|  |  | 5% | >10^24^ | >10^24^ | >10^24^ | >10^24^ | >10^24^ | >10^24^ |  | 24.058 | >10^24^ | >10^24^ | >10^24^ | >10^24^ | 0.224 |
|  | $\pi^{*}$ | 50% | 0.183 | 0.198 | 0.219 | 0.237 | >10^24^ | >10^24^ |  | 0.160 | 0.177 | 0.195 | 0.213 | 0.496 | 4.469 |
|  |  | 10% | 1.932 | 0.531 | 0.318 | 0.263 | 0.204 | 0.189 |  | 1.812 | 0.494 | 0.314 | 0.257 | 0.181 | 0.171 |
|  |  | 5% | 20.481 | 7.765 | 3.034 | 1.485 | 0.334 | 0.247 |  | 18.699 | 6.801 | 2.951 | 1.467 | 0.335 | 0.215 |
| Coverage | $\pi$ | 50% | 91.6 | 91.2 | 90.7 | 90.7 | 87.9 | 84.7 |  | 89.3 | 90.2 | 91.0 | 91.4 | 92.7 | 93.0 |
| (%) |  | 10% | 85.9 | 87.7 | 89.0 | 89.5 | 91.0 | 91.8 |  | 91.0 | 91.1 | 91.1 | 91.1 | 91.6 | 91.3 |
|  |  | 5% | 79.7 | 84.0 | 86.0 | 86.8 | 89.2 | 90.7 |  | 85.8 | 89.4 | 91.0 | 91.4 | 91.4 | 92.1 |
|  | $\pi^{*}$ | 50% | 93.3 | 93.6 | 93.3 | 93.3 | 92.7 | 92.9 |  | 93.6 | 93.2 | 93.2 | 93.3 | 93.2 | 93.7 |
|  |  | 10% | 93.8 | 93.5 | 93.6 | 93.5 | 93.7 | 93.8 |  | 94.1 | 93.9 | 93.7 | 93.7 | 93.9 | 93.9 |
|  |  | 5% | 88.1 | 93.1 | 94.0 | 93.7 | 93.5 | 93.3 |  | 88.9 | 93.5 | 94.0 | 94.2 | 93.8 | 94.0 |
| Type I error | $\pi$ | 50% | 8.4 | 18.5 | 33.6 | 47.6 | 78.5 | 90.8 |  | 10.6 | 42.2 | 69.8 | 85.3 | 99.1 | 99.9 |
| and power |  | 10% | 14.1 | 13.8 | 23.5 | 36.1 | 82.1 | 97.9 |  | 9.0 | 27.1 | 49.3 | 69.1 | 98.4 | 100.0 |
| (%) |  | 5% | 20.2 | 16.2 | 19.6 | 27.8 | 68.9 | 95.0 |  | 14.2 | 22.1 | 36.7 | 51.5 | 91.6 | 99.8 |
|  | $\pi^{*}$ | 50% | 6.7 | 25.2 | 48.4 | 66.0 | 92.0 | 96.8 |  | 6.4 | 26.8 | 52.9 | 69.9 | 94.3 | 98.3 |
|  |  | 10% | 6.1 | 16.7 | 35.8 | 54.6 | 94.8 | 99.6 |  | 5.9 | 18.2 | 38.7 | 58.1 | 96.7 | 99.8 |
|  |  | 5% | 11.9 | 15.4 | 26.9 | 40.9 | 86.1 | 99.0 |  | 11.1 | 15.5 | 28.2 | 43.1 | 87.8 | 99.4 |

[Note] Scenario I: the situation wherein the covariate distribution is similar between the randomized control trial (RCT) data and historical control data; Scenario II: the situation wherein the covariate distribution is not similar between the RCT data and historical control data; $\pi$ (without $X_{r}$): the conventional method; $\pi^{*}$ (with $X_{r}$): the proposed method.


Performance of the estimated propensity score (PS) model by simulation setting assuming Rt:Rc:Hc = 2:1:27

|  |  |  | **Scenario Ⅰ** | | | | | |  | **Scenario Ⅱ** | | | | | |
| --- | --- | --- | --- | --- | --- | --- | --- | --- | --- | --- | --- | --- | --- | --- | --- |
|  |  |  | Odds ratio | | | | | |  | Odds ratio | | | | | |
| Performance measurement | PS model | Outcome event rate | 1.0 | 1.5 | 2.0 | 2.5 | 5.0 | 10.0 |  | 1.0 | 1.5 | 2.0 | 2.5 | 5.0 | 10.0 |
| Bias | $\pi$ | 50% | 0.006 | 0.018 | 0.033 | 0.044 | >10^9^ | >10^9^ |  | 0.247 | 0.247 | 0.250 | 0.254 | 0.278 | 0.380 |
|  | (without $X_{r}$) | 10% | <-10^9^ | <-10^9^ | <-10^9^ | -0.102 | -0.072 | -0.052 |  | <-10^9^ | <-10^9^ | <-10^9^ | <-10^9^ | 0.185 | 0.195 |
|  |  | 5% | <-10^9^ | <-10^9^ | <-10^9^ | <-10^9^ | <-10^9^ | -0.077 |  | -0.535 | -0.077 | <-10^9^ | <-10^9^ | <-10^9^ | 0.191 |
|  | $\pi^{*}$ | 50% | 0.088 | 0.087 | 0.092 | 0.096 | 0.116 | 0.213 |  | 0.076 | 0.075 | 0.078 | 0.078 | 0.096 | 0.182 |
|  | (with $X_{r}$) | 10% | -0.022 | 0.016 | 0.030 | 0.035 | 0.043 | 0.042 |  | -0.017 | 0.021 | 0.031 | 0.036 | 0.040 | 0.035 |
|  |  | 5% | -0.566 | -0.151 | -0.045 | -0.011 | 0.039 | 0.055 |  | -0.551 | -0.161 | -0.037 | -0.005 | 0.036 | 0.049 |
| MSE | $\pi$ | 50% | 0.223 | 0.237 | 0.256 | 0.285 | >10^24^ | >10^24^ |  | 0.173 | 0.179 | 0.192 | 0.206 | 0.360 | 1.457 |
|  |  | 10% | >10^24^ | >10^24^ | >10^24^ | 0.338 | 0.263 | 0.240 |  | >10^24^ | >10^24^ | >10^24^ | >10^24^ | 0.174 | 0.166 |
|  |  | 5% | >10^24^ | >10^24^ | >10^24^ | >10^24^ | >10^24^ | 0.311 |  | 10.965 | 3.104 | >10^24^ | >10^24^ | >10^24^ | 0.215 |
|  | $\pi^{*}$ | 50% | 0.113 | 0.121 | 0.131 | 0.146 | 0.214 | 1.200 |  | 0.100 | 0.108 | 0.117 | 0.128 | 0.244 | 1.083 |
|  |  | 10% | 0.561 | 0.298 | 0.187 | 0.166 | 0.136 | 0.126 |  | 0.483 | 0.204 | 0.173 | 0.155 | 0.122 | 0.117 |
|  |  | 5% | 8.915 | 2.368 | 0.874 | 0.567 | 0.210 | 0.175 |  | 8.523 | 2.407 | 0.773 | 0.442 | 0.197 | 0.164 |
| Coverage | $\pi$ | 50% | 92.2 | 91.9 | 91.4 | 89.9 | 87.1 | 93.2 |  | 87.7 | 88.4 | 88.8 | 89.6 | 90.7 | 91.7 |
| (%) |  | 10% | 89.6 | 90.4 | 91.0 | 91.9 | 92.2 | 94.7 |  | 90.9 | 90.4 | 90.9 | 90.6 | 90.9 | 90.9 |
|  |  | 5% | 86.8 | 88.0 | 88.9 | 90.6 | 92.0 | 94.4 |  | 88.4 | 90.3 | 90.7 | 91.0 | 91.3 | 91.3 |
|  | $\pi^{*}$ | 50% | 93.8 | 93.8 | 94.0 | 93.9 | 94.3 | 95.3 |  | 94.8 | 94.3 | 94.6 | 94.6 | 94.6 | 95.3 |
|  |  | 10% | 94.8 | 94.1 | 94.0 | 93.6 | 93.6 | 94.3 |  | 94.8 | 94.4 | 94.0 | 94.1 | 94.6 | 94.7 |
|  |  | 5% | 92.3 | 94.5 | 94.6 | 94.5 | 94.0 | 94.0 |  | 92.5 | 94.7 | 94.8 | 94.7 | 94.1 | 94.5 |
| Type I error | $\pi$ | 50% | 7.5 | 20.1 | 39.6 | 55.9 | 86.2 | 94.6 |  | 12.2 | 48.4 | 77.6 | 91.1 | 99.6 | 99.9 |
| and power |  | 10% | 11.4 | 13.9 | 26.6 | 42.6 | 89.4 | 99.3 |  | 9.0 | 29.8 | 55.6 | 75.3 | 99.3 | 100.0 |
| (%) |  | 5% | 15.8 | 13.8 | 20.6 | 31.5 | 77.5 | 98.0 |  | 11.6 | 21.9 | 39.2 | 56.2 | 94.6 | 99.9 |
|  | $\pi^{*}$ | 50% | 6.2 | 35.1 | 67.0 | 83.9 | 98.2 | 99.4 |  | 5.2 | 34.4 | 68.0 | 85.7 | 99.0 | 99.7 |
|  |  | 10% | 5.2 | 22.9 | 48.3 | 70.3 | 98.5 | 99.9 |  | 5.1 | 23.0 | 50.1 | 71.7 | 99.0 | 100.0 |
|  |  | 5% | 7.7 | 16.8 | 34.0 | 51.9 | 93.5 | 99.8 |  | 7.5 | 16.8 | 34.6 | 52.8 | 94.4 | 99.8 |

[Note] Scenario I: the situation wherein the covariate distribution is similar between the randomized control trial (RCT) data and historical control data; Scenario II: the situation wherein the covariate distribution is not similar between the RCT data and historical control data; $\pi$ (without $X_{r}$): the conventional method; $\pi^{*}$ (with $X_{r}$): the proposed method.


Performance of the estimated propensity score (PS) model by simulation setting assuming Rt:Rc:Hc = 9:1:90

|  |  |  | **Scenario Ⅰ** | | | | | |  | **Scenario Ⅱ** | | | | | |
| --- | --- | --- | --- | --- | --- | --- | --- | --- | --- | --- | --- | --- | --- | --- | --- |
|  |  |  | Odds ratio | | | | | |  | Odds ratio | | | | | |
| Performance measurement | PS model | Outcome event rate | 1.0 | 1.5 | 2.0 | 2.5 | 5.0 | 10.0 |  | 1.0 | 1.5 | 2.0 | 2.5 | 5.0 | 10.0 |
| Bias | $\pi$ | 50% | 0.000 | 0.004 | 0.008 | 0.014 | >10^9^ | >10^9^ |  | 0.270 | 0.270 | 0.272 | 0.276 | 0.290 | 0.334 |
|  | (without $X_{r}$) | 10% | <-10^9^ | <-10^9^ | -0.078 | -0.070 | -0.051 | -0.047 |  | <-10^9^ | <-10^9^ | <-10^9^ | <-10^9^ | 0.207 | 0.217 |
|  |  | 5% | <-10^9^ | <-10^9^ | <-10^9^ | <-10^9^ | <-10^9^ | -0.052 |  | <-10^9^ | <-10^9^ | <-10^9^ | <-10^9^ | <-10^9^ | 0.211 |
|  | $\pi^{*}$ | 50% | 0.107 | 0.104 | 0.102 | 0.103 | 0.105 | 0.134 |  | 0.079 | 0.073 | 0.074 | 0.072 | 0.071 | 0.088 |
|  | (with $X_{r}$) | 10% | 0.071 | 0.088 | 0.093 | 0.095 | 0.097 | 0.086 |  | 0.040 | 0.056 | 0.062 | 0.063 | 0.062 | 0.058 |
|  |  | 5% | -0.117 | 0.039 | 0.068 | 0.081 | 0.104 | 0.117 |  | -0.163 | 0.000 | 0.041 | 0.056 | 0.076 | 0.086 |
| MSE | $\pi$ | 50% | 0.157 | 0.166 | 0.182 | 0.197 | >10^24^ | >10^24^ |  | 0.189 | 0.194 | 0.204 | 0.217 | 0.282 | 0.448 |
|  |  | 10% | >10^24^ | >10^24^ | 0.266 | 0.239 | 0.193 | 0.176 |  | >10^24^ | >10^24^ | >10^24^ | >10^24^ | 0.192 | 0.180 |
|  |  | 5% | >10^24^ | >10^24^ | >10^24^ | >10^24^ | >10^24^ | 0.242 |  | >10^24^ | >10^24^ | >10^24^ | >10^24^ | >10^24^ | 0.244 |
|  | $\pi^{*}$ | 50% | 0.085 | 0.088 | 0.092 | 0.099 | 0.131 | 0.291 |  | 0.076 | 0.079 | 0.083 | 0.088 | 0.118 | 0.194 |
|  |  | 10% | 0.206 | 0.164 | 0.149 | 0.139 | 0.120 | 0.115 |  | 0.192 | 0.156 | 0.139 | 0.129 | 0.110 | 0.107 |
|  |  | 5% | 2.802 | 0.599 | 0.309 | 0.233 | 0.188 | 0.177 |  | 2.970 | 0.628 | 0.300 | 0.218 | 0.178 | 0.166 |
| Coverage | $\pi$ | 50% | 93.1 | 93.0 | 92.9 | 92.4 | 91.2 | 89.6 |  | 85.5 | 85.8 | 86.0 | 86.2 | 87.7 | 88.1 |
| (%) |  | 10% | 89.9 | 91.3 | 91.8 | 91.7 | 92.5 | 93.1 |  | 90.0 | 89.8 | 89.9 | 90.0 | 90.0 | 89.7 |
|  |  | 5% | 87.3 | 89.1 | 90.0 | 90.5 | 91.8 | 92.7 |  | 88.0 | 89.4 | 90.2 | 90.4 | 90.2 | 90.9 |
|  | $\pi^{*}$ | 50% | 93.0 | 93.2 | 94.1 | 94.2 | 95.0 | 96.1 |  | 94.4 | 94.5 | 94.7 | 94.9 | 95.4 | 96.2 |
|  |  | 10% | 92.9 | 92.4 | 92.3 | 92.2 | 92.2 | 93.3 |  | 93.9 | 93.1 | 93.0 | 93.0 | 93.3 | 93.6 |
|  |  | 5% | 93.0 | 93.3 | 93.0 | 92.5 | 92.2 | 92.5 |  | 93.4 | 93.8 | 93.5 | 93.4 | 92.7 | 93.4 |
| Type I error | $\pi$ | 50% | 6.9 | 22.9 | 47.5 | 65.6 | 92.0 | 96.9 |  | 14.5 | 53.2 | 79.7 | 91.2 | 99.0 | 99.7 |
| and power |  | 10% | 10.0 | 14.4 | 32.6 | 52.8 | 95.6 | 99.8 |  | 10.0 | 30.8 | 56.8 | 76.7 | 99.3 | 100.0 |
| (%) |  | 5% | 12.7 | 13.3 | 23.7 | 37.3 | 86.8 | 99.3 |  | 12.0 | 22.3 | 39.9 | 57.3 | 95.2 | 99.9 |
|  | $\pi^{*}$ | 50% | 7.0 | 48.9 | 83.5 | 94.8 | 99.6 | 99.8 |  | 5.6 | 45.1 | 81.9 | 94.8 | 99.8 | 99.9 |
|  |  | 10% | 7.0 | 34.2 | 64.5 | 83.2 | 98.3 | 99.5 |  | 6.0 | 30.5 | 61.9 | 81.1 | 98.6 | 99.6 |
|  |  | 5% | 7.0 | 24.2 | 47.4 | 66.0 | 95.7 | 98.9 |  | 6.6 | 21.9 | 44.7 | 64.4 | 95.5 | 99.2 |

[Note] Scenario I: the situation wherein the covariate distribution is similar between the randomized control trial (RCT) data and historical control data; Scenario II: the situation wherein the covariate distribution is not similar between the RCT data and historical control data; $\pi$ (without $X_{r}$): the conventional method; $\pi^{*}$ (with $X_{r}$): the proposed method.


Performance of the estimated propensity score (PS) model by simulation setting assuming Rt:Rc:Hc = 2:1:1

|  |  |  | **Scenario Ⅰ** | | | | | |  | **Scenario Ⅱ** | | | | | |
| --- | --- | --- | --- | --- | --- | --- | --- | --- | --- | --- | --- | --- | --- | --- | --- |
|  |  |  | Odds ratio | | | | | |  | Odds ratio | | | | | |
| Performance measurement | PS model | Outcome event rate | 1.0 | 1.5 | 2.0 | 2.5 | 5.0 | 10.0 |  | 1.0 | 1.5 | 2.0 | 2.5 | 5.0 | 10.0 |
| Bias | $\pi$ | 50% | -0.002 | -0.011 | -0.018 | -0.023 | -0.041 | -0.057 |  | 0.135 | 0.123 | 0.116 | 0.111 | 0.094 | 0.078 |
|  | (without $X_{r}$) | 10% | 0.003 | 0.005 | 0.006 | 0.009 | 0.022 | 0.063 |  | 0.130 | 0.132 | 0.132 | 0.132 | 0.143 | 0.173 |
|  |  | 5% | 0.004 | 0.015 | 0.026 | 0.037 | 0.099 | 0.432 |  | 0.128 | 0.135 | 0.145 | 0.151 | 0.203 | 0.513 |
|  | $\pi^{*}$ | 50% | 0.018 | 0.009 | 0.002 | -0.002 | -0.017 | -0.034 |  | 0.023 | 0.012 | 0.005 | 0.003 | -0.016 | -0.031 |
|  | (with $X_{r}$) | 10% | 0.034 | 0.038 | 0.041 | 0.045 | 0.068 | 0.120 |  | 0.031 | 0.038 | 0.042 | 0.046 | 0.071 | 0.129 |
|  |  | 5% | 0.045 | 0.061 | 0.076 | 0.089 | 0.167 | 0.531 |  | 0.043 | 0.058 | 0.074 | 0.085 | 0.170 | 0.531 |
| MSE | $\pi$ | 50% | 0.026 | 0.027 | 0.028 | 0.029 | 0.033 | 0.040 |  | 0.035 | 0.033 | 0.032 | 0.031 | 0.030 | 0.031 |
|  |  | 10% | 0.074 | 0.079 | 0.085 | 0.092 | 0.132 | 0.261 |  | 0.067 | 0.071 | 0.076 | 0.081 | 0.113 | 0.247 |
|  |  | 5% | 0.145 | 0.157 | 0.170 | 0.189 | 0.553 | 5.212 |  | 0.117 | 0.127 | 0.140 | 0.155 | 0.575 | 5.039 |
|  | $\pi^{*}$ | 50% | 0.031 | 0.031 | 0.032 | 0.033 | 0.037 | 0.045 |  | 0.030 | 0.030 | 0.031 | 0.033 | 0.037 | 0.044 |
|  |  | 10% | 0.090 | 0.100 | 0.111 | 0.122 | 0.183 | 0.348 |  | 0.089 | 0.099 | 0.111 | 0.122 | 0.183 | 0.379 |
|  |  | 5% | 0.174 | 0.198 | 0.221 | 0.249 | 0.662 | 5.473 |  | 0.173 | 0.194 | 0.219 | 0.247 | 0.749 | 5.380 |
| Coverage | $\pi$ | 50% | 95.2 | 95.1 | 94.9 | 94.7 | 94.4 | 94.0 |  | 83.9 | 86.0 | 87.1 | 88.3 | 91.1 | 93.0 |
| (%) |  | 10% | 94.6 | 94.5 | 94.5 | 94.3 | 94.0 | 93.6 |  | 91.6 | 92.2 | 92.7 | 93.1 | 94.6 | 96.0 |
|  |  | 5% | 94.3 | 94.3 | 94.3 | 94.2 | 94.0 | 93.9 |  | 93.7 | 94.0 | 94.6 | 95.1 | 96.5 | 96.4 |
|  | $\pi^{*}$ | 50% | 94.7 | 94.8 | 95.0 | 94.7 | 94.9 | 94.6 |  | 95.0 | 94.7 | 94.9 | 94.6 | 94.8 | 94.4 |
|  |  | 10% | 93.8 | 93.6 | 93.2 | 92.6 | 92.0 | 90.9 |  | 93.7 | 93.5 | 92.9 | 92.7 | 91.8 | 90.5 |
|  |  | 5% | 93.4 | 92.4 | 92.4 | 91.8 | 90.5 | 89.4 |  | 93.0 | 92.6 | 92.4 | 91.9 | 90.4 | 89.8 |
| Type I error | $\pi$ | 50% | 4.8 | 67.7 | 98.2 | 99.9 | 100.0 | 100.0 |  | 16.0 | 97.6 | 100.0 | 100.0 | 100.0 | 100.0 |
| and power |  | 10% | 5.3 | 32.3 | 69.7 | 88.2 | 99.4 | 99.8 |  | 8.3 | 63.9 | 94.3 | 99.3 | 100.0 | 100.0 |
| (%) |  | 5% | 5.7 | 21.3 | 46.0 | 66.1 | 94.6 | 98.6 |  | 6.3 | 37.1 | 71.2 | 88.6 | 99.9 | 100.0 |
|  | $\pi^{*}$ | 50% | 5.3 | 65.8 | 96.8 | 99.7 | 100.0 | 100.0 |  | 5.0 | 67.6 | 97.3 | 99.8 | 100.0 | 100.0 |
|  |  | 10% | 6.2 | 34.4 | 65.7 | 82.7 | 97.6 | 99.2 |  | 6.3 | 34.5 | 65.6 | 82.8 | 97.9 | 99.3 |
|  |  | 5% | 6.6 | 24.6 | 47.0 | 63.1 | 90.0 | 96.6 |  | 7.0 | 24.2 | 46.7 | 62.7 | 89.6 | 96.3 |

[Note] Scenario I: the situation wherein the covariate distribution is similar between the randomized control trial (RCT) data and historical control data; Scenario II: the situation wherein the covariate distribution is not similar between the RCT data and historical control data; $\pi$ (without $X_{r}$): the conventional method; $\pi^{*}$ (with $X_{r}$): the proposed method.


Performance of the estimated propensity score (PS) model by simulation setting assuming Rt:Rc:Hc = 3:1:2

|  |  |  | **Scenario Ⅰ** | | | | | |  | **Scenario Ⅱ** | | | | | |
| --- | --- | --- | --- | --- | --- | --- | --- | --- | --- | --- | --- | --- | --- | --- | --- |
|  |  |  | Odds ratio | | | | | |  | Odds ratio | | | | | |
| Performance measurement | PS model | Outcome event rate | 1.0 | 1.5 | 2.0 | 2.5 | 5.0 | 10.0 |  | 1.0 | 1.5 | 2.0 | 2.5 | 5.0 | 10.0 |
| Bias | $\pi$ | 50% | -0.002 | -0.011 | -0.018 | -0.023 | -0.041 | -0.057 |  | 0.163 | 0.152 | 0.144 | 0.138 | 0.120 | 0.105 |
|  | (without $X_{r}$) | 10% | 0.003 | 0.005 | 0.006 | 0.009 | 0.022 | 0.063 |  | 0.159 | 0.162 | 0.163 | 0.163 | 0.172 | 0.203 |
|  |  | 5% | 0.004 | 0.015 | 0.026 | 0.037 | 0.099 | 0.432 |  | 0.158 | 0.166 | 0.176 | 0.184 | 0.228 | 0.555 |
|  | $\pi^{*}$ | 50% | 0.018 | 0.028 | 0.020 | 0.014 | 0.010 | -0.005 |  | 0.031 | 0.020 | 0.013 | 0.009 | -0.008 | -0.023 |
|  | (with $X_{r}$) | 10% | 0.034 | 0.053 | 0.059 | 0.062 | 0.068 | 0.100 |  | 0.048 | 0.057 | 0.063 | 0.070 | 0.099 | 0.172 |
|  |  | 5% | 0.045 | 0.072 | 0.091 | 0.110 | 0.126 | 0.219 |  | 0.071 | 0.088 | 0.107 | 0.124 | 0.213 | 0.603 |
| MSE | $\pi$ | 50% | 0.026 | 0.027 | 0.028 | 0.029 | 0.033 | 0.040 |  | 0.044 | 0.041 | 0.040 | 0.038 | 0.036 | 0.036 |
|  |  | 10% | 0.074 | 0.079 | 0.085 | 0.092 | 0.132 | 0.261 |  | 0.078 | 0.083 | 0.087 | 0.093 | 0.128 | 0.266 |
|  |  | 5% | 0.145 | 0.157 | 0.170 | 0.189 | 0.553 | 5.212 |  | 0.131 | 0.142 | 0.157 | 0.176 | 0.464 | 5.270 |
|  | $\pi^{*}$ | 50% | 0.038 | 0.038 | 0.039 | 0.039 | 0.044 | 0.053 |  | 0.036 | 0.037 | 0.039 | 0.040 | 0.044 | 0.052 |
|  |  | 10% | 0.109 | 0.122 | 0.137 | 0.152 | 0.230 | 0.423 |  | 0.110 | 0.124 | 0.139 | 0.156 | 0.232 | 0.459 |
|  |  | 5% | 0.205 | 0.238 | 0.270 | 0.307 | 0.751 | 5.687 |  | 0.209 | 0.238 | 0.272 | 0.310 | 0.712 | 5.755 |
| Coverage | $\pi$ | 50% | 95.2 | 95.1 | 94.9 | 94.7 | 94.4 | 94.0 |  | 78.0 | 80.7 | 82.8 | 84.2 | 88.4 | 90.6 |
| (%) |  | 10% | 94.6 | 94.5 | 94.5 | 94.3 | 94.0 | 93.6 |  | 89.7 | 90.2 | 90.9 | 91.4 | 93.3 | 95.0 |
|  |  | 5% | 94.3 | 94.3 | 94.3 | 94.2 | 94.0 | 93.9 |  | 92.8 | 92.9 | 93.3 | 93.5 | 95.6 | 96.4 |
|  | $\pi^{*}$ | 50% | 94.4 | 94.2 | 94.6 | 94.4 | 94.7 | 94.7 |  | 94.9 | 94.8 | 94.5 | 94.3 | 94.8 | 94.4 |
|  |  | 10% | 92.3 | 92.0 | 91.4 | 91.0 | 89.3 | 87.0 |  | 92.4 | 91.8 | 91.3 | 90.9 | 89.5 | 86.4 |
|  |  | 5% | 91.9 | 90.6 | 89.9 | 89.0 | 86.6 | 86.3 |  | 91.2 | 90.6 | 89.5 | 88.9 | 86.4 | 86.4 |
| Type I error | $\pi$ | 50% | 4.8 | 67.7 | 98.2 | 99.9 | 100.0 | 100.0 |  | 21.9 | 98.4 | 100.0 | 100.0 | 100.0 | 100.0 |
| and power |  | 10% | 5.3 | 32.3 | 69.7 | 88.2 | 99.4 | 99.8 |  | 10.3 | 67.8 | 94.8 | 99.1 | 100.0 | 100.0 |
| (%) |  | 5% | 5.7 | 21.3 | 46.0 | 66.1 | 94.6 | 98.6 |  | 7.1 | 40.3 | 72.6 | 89.1 | 99.8 | 100.0 |
|  | $\pi^{*}$ | 50% | 5.6 | 61.4 | 94.3 | 99.0 | 100.0 | 100.0 |  | 5.1 | 61.8 | 94.5 | 99.2 | 100.0 | 100.0 |
|  |  | 10% | 7.7 | 34.5 | 61.8 | 77.5 | 95.8 | 98.5 |  | 7.6 | 34.8 | 62.0 | 78.1 | 95.6 | 98.5 |
|  |  | 5% | 8.1 | 26.7 | 46.8 | 61.2 | 86.6 | 94.3 |  | 8.8 | 26.3 | 46.6 | 60.8 | 87.1 | 94.5 |

[Note] Scenario I: the situation wherein the covariate distribution is similar between the randomized control trial (RCT) data and historical control data; Scenario II: the situation wherein the covariate distribution is not similar between the RCT data and historical control data; $\pi$ (without $X_{r}$): the conventional method; $\pi^{*}$ (with $X_{r}$): the proposed method.

1. **Simulation setting assuming that one of the covariates is binary data**

The text presented the simulation result by setting that four covariates are generated from the multivariate standard normal distribution. In this chapter, the case of setting that a binary covariate was also examined. The simulation results assuming that one of the four covariates was binary data generated from the bernoulli distribution are shown below. The method and conditions in the simulation setting are the same as those shown in the text, except for the method of generated of $X_{i4}$.

From the multivariate standard normal distribution, three covariates are generated for participant$i$ as

$$\begin{aligned} \left\{ X_{i1},X_{i2},X_{i3} \right\}\sim N\left( 0, 1 \right),\#\left( C.1 \right) \end{aligned}$$

and one covariate is generated for participant$i$ as

$$\begin{aligned} \left\{ X_{i4} \right\}\sim Bernoulli\left( 0.50 \right).\#\left( C.2 \right) \end{aligned}$$

Performance of the estimated propensity score (PS) model by simulation setting assuming three continuous covariates and one binary covariate

|  |  |  | **Scenario Ⅰ** | | | | | |  | **Scenario Ⅱ** | | | | | |
| --- | --- | --- | --- | --- | --- | --- | --- | --- | --- | --- | --- | --- | --- | --- | --- |
|  |  |  | Odds ratio | | | | | |  | Odds ratio | | | | | |
| Performance measurement | PS model | Outcome event rate | 1.0 | 1.5 | 2.0 | 2.5 | 5.0 | 10.0 |  | 1.0 | 1.5 | 2.0 | 2.5 | 5.0 | 10.0 |
| Bias | $\pi$ | 50% | 0.002 | -0.007 | -0.014 | -0.019 | -0.035 | -0.047 |  | 0.169 | 0.158 | 0.154 | 0.150 | 0.141 | 0.137 |
|  | (without $X_{r}$) | 10% | 0.002 | 0.003 | 0.008 | 0.011 | 0.028 | 0.072 |  | 0.151 | 0.153 | 0.152 | 0.153 | 0.151 | 0.154 |
|  |  | 5% | 0.011 | 0.021 | 0.034 | 0.044 | 0.109 | 0.483 |  | 0.134 | 0.146 | 0.153 | 0.157 | 0.170 | 0.188 |
|  | $\pi^{*}$ | 50% | 0.034 | 0.027 | 0.019 | 0.017 | 0.000 | -0.009 |  | 0.044 | 0.030 | 0.026 | 0.023 | 0.014 | 0.010 |
|  | (with $X_{r}$) | 10% | 0.057 | 0.062 | 0.069 | 0.075 | 0.106 | 0.180 |  | 0.030 | 0.034 | 0.034 | 0.035 | 0.035 | 0.042 |
|  |  | 5% | 0.085 | 0.100 | 0.120 | 0.138 | 0.232 | 0.656 |  | 0.009 | 0.026 | 0.035 | 0.042 | 0.063 | 0.094 |
| MSE | $\pi$ | 50% | 0.026 | 0.026 | 0.027 | 0.028 | 0.032 | 0.039 |  | 0.052 | 0.051 | 0.051 | 0.051 | 0.054 | 0.067 |
|  |  | 10% | 0.076 | 0.081 | 0.088 | 0.096 | 0.139 | 0.297 |  | 0.090 | 0.083 | 0.079 | 0.078 | 0.079 | 0.093 |
|  |  | 5% | 0.147 | 0.158 | 0.174 | 0.195 | 0.540 | 5.786 |  | 0.152 | 0.136 | 0.131 | 0.129 | 0.143 | 0.187 |
|  | $\pi^{*}$ | 50% | 0.037 | 0.036 | 0.038 | 0.039 | 0.045 | 0.052 |  | 0.035 | 0.038 | 0.039 | 0.040 | 0.050 | 0.069 |
|  |  | 10% | 0.112 | 0.124 | 0.139 | 0.155 | 0.231 | 0.464 |  | 0.095 | 0.085 | 0.081 | 0.080 | 0.083 | 0.103 |
|  |  | 5% | 0.210 | 0.239 | 0.270 | 0.310 | 0.743 | 6.274 |  | 0.179 | 0.156 | 0.148 | 0.147 | 0.163 | 0.220 |
| Coverage | $\pi$ | 50% | 95.5 | 95.3 | 95.3 | 94.9 | 94.8 | 94.5 |  | 82.0 | 83.6 | 84.9 | 86.3 | 89.8 | 92.5 |
| (%) |  | 10% | 94.3 | 94.2 | 94.2 | 93.8 | 93.8 | 93.7 |  | 89.2 | 89.3 | 89.3 | 89.4 | 90.8 | 92.5 |
|  |  | 5% | 94.1 | 93.9 | 93.9 | 94.0 | 93.6 | 94.0 |  | 92.3 | 92.3 | 92.2 | 92.4 | 93.1 | 94.7 |
|  | $\pi^{*}$ | 50% | 94.5 | 94.8 | 94.5 | 94.7 | 94.9 | 95.0 |  | 95.0 | 94.5 | 94.6 | 94.9 | 94.9 | 94.8 |
|  |  | 10% | 92.4 | 92.1 | 91.6 | 91.3 | 89.4 | 86.9 |  | 93.7 | 94.1 | 94.0 | 93.9 | 94.0 | 94.0 |
|  |  | 5% | 91.3 | 90.7 | 89.8 | 88.7 | 86.1 | 86.0 |  | 94.2 | 94.4 | 94.2 | 94.0 | 93.8 | 93.9 |
| Type I error | $\pi$ | 50% | 4.5 | 68.8 | 98.4 | 99.9 | 100.0 | 100.0 |  | 18.0 | 94.5 | 100.0 | 100.0 | 100.0 | 100.0 |
| and power |  | 10% | 5.6 | 33.1 | 69.5 | 87.9 | 99.3 | 99.8 |  | 10.7 | 63.7 | 93.7 | 99.3 | 100.0 | 100.0 |
| (%) |  | 5% | 5.9 | 21.5 | 46.9 | 66.3 | 94.1 | 98.3 |  | 7.7 | 42.0 | 74.4 | 90.8 | 100.0 | 100.0 |
|  | $\pi^{*}$ | 50% | 5.5 | 62.9 | 94.7 | 99.3 | 99.9 | 100.0 |  | 5.0 | 63.8 | 95.8 | 99.5 | 100.0 | 100.0 |
|  |  | 10% | 7.6 | 35.4 | 62.4 | 78.1 | 95.4 | 98.3 |  | 6.3 | 36.8 | 74.9 | 92.5 | 99.9 | 100.0 |
|  |  | 5% | 8.6 | 27.6 | 47.6 | 62.0 | 86.9 | 94.5 |  | 5.8 | 24.0 | 52.3 | 73.7 | 98.6 | 99.8 |

[Note] Scenario I: the situation wherein the covariate distribution is similar between the randomized control trial (RCT) data and historical control data; Scenario II: the situation wherein the covariate distribution is not similar between the RCT data and historical control data; $\pi$ (without $X_{r}$): the conventional method; $\pi^{*}$ (with $X_{r}$): the proposed method.

1. **Simulation setting assuming that the randomized assignment of treatment variables**

In this chapter, the case of setting that the assignment of treatment variables was completely random in the RCT population was also examined. The simulation results that assume a random assignment of treatment variables according to an allocation ratio between the RCT treatment group, RCT control group, and historical control group as 1:1:2 are shown below. The method and conditions in the simulation setting are the same as those shown in the text, except for the method of the assignment of treatment variables.

Performance of the estimated propensity score (PS) model by simulation setting assuming the randomized assignment of treatment variables

|  |  |  | **Scenario Ⅰ** | | | | | |  | **Scenario Ⅱ** | | | | | |
| --- | --- | --- | --- | --- | --- | --- | --- | --- | --- | --- | --- | --- | --- | --- | --- |
|  |  |  | Odds ratio | | | | | |  | Odds ratio | | | | | |
| Performance measurement | PS model | Outcome event rate | 1.0 | 1.5 | 2.0 | 2.5 | 5.0 | 10.0 |  | 1.0 | 1.5 | 2.0 | 2.5 | 5.0 | 10.0 |
| Bias | $\pi$ | 50% | -0.001 | -0.013 | -0.020 | -0.024 | -0.039 | -0.047 |  | 0.153 | 0.143 | 0.136 | 0.131 | 0.120 | 0.118 |
|  | (without $X_{r}$) | 10% | -0.014 | -0.013 | -0.014 | -0.014 | -0.016 | -0.014 |  | 0.135 | 0.135 | 0.136 | 0.136 | 0.136 | 0.138 |
|  |  | 5% | -0.032 | -0.021 | -0.015 | -0.010 | 0.003 | 0.019 |  | 0.111 | 0.126 | 0.133 | 0.138 | 0.152 | 0.172 |
|  | $\pi^{*}$ | 50% | -0.001 | -0.013 | -0.020 | -0.024 | -0.039 | -0.047 |  | 0.040 | 0.030 | 0.022 | 0.017 | 0.004 | 0.000 |
|  | (with $X_{r}$) | 10% | -0.013 | -0.012 | -0.013 | -0.013 | -0.015 | -0.011 |  | 0.029 | 0.030 | 0.029 | 0.029 | 0.028 | 0.030 |
|  |  | 5% | -0.031 | -0.019 | -0.012 | -0.007 | 0.006 | 0.024 |  | 0.012 | 0.025 | 0.030 | 0.035 | 0.047 | 0.068 |
| MSE | $\pi$ | 50% | 0.023 | 0.024 | 0.025 | 0.026 | 0.034 | 0.047 |  | 0.052 | 0.049 | 0.049 | 0.048 | 0.054 | 0.068 |
|  |  | 10% | 0.070 | 0.060 | 0.056 | 0.055 | 0.054 | 0.063 |  | 0.100 | 0.089 | 0.085 | 0.082 | 0.081 | 0.092 |
|  |  | 5% | 0.145 | 0.121 | 0.110 | 0.106 | 0.106 | 0.137 |  | 0.182 | 0.155 | 0.145 | 0.140 | 0.145 | 0.184 |
|  | $\pi^{*}$ | 50% | 0.024 | 0.025 | 0.026 | 0.027 | 0.035 | 0.047 |  | 0.026 | 0.026 | 0.026 | 0.027 | 0.034 | 0.046 |
|  |  | 10% | 0.072 | 0.063 | 0.059 | 0.058 | 0.058 | 0.069 |  | 0.071 | 0.063 | 0.060 | 0.058 | 0.059 | 0.070 |
|  |  | 5% | 0.150 | 0.126 | 0.116 | 0.112 | 0.115 | 0.151 |  | 0.144 | 0.122 | 0.114 | 0.110 | 0.118 | 0.159 |
| Coverage | $\pi$ | 50% | 95.3 | 95.2 | 94.9 | 95.1 | 93.9 | 93.5 |  | 85.4 | 87.4 | 88.4 | 89.6 | 91.0 | 92.7 |
| (%) |  | 10% | 95.6 | 95.5 | 95.4 | 95.2 | 95.5 | 95.2 |  | 90.5 | 91.1 | 91.1 | 91.2 | 91.7 | 92.7 |
|  |  | 5% | 95.5 | 95.2 | 95.5 | 95.4 | 95.4 | 95.4 |  | 92.4 | 92.3 | 92.5 | 92.4 | 93.5 | 94.5 |
|  | $\pi^{*}$ | 50% | 95.4 | 95.1 | 94.9 | 95.2 | 94.0 | 93.7 |  | 94.6 | 94.8 | 95.2 | 95.3 | 95.0 | 95.1 |
|  |  | 10% | 95.0 | 95.4 | 95.2 | 95.0 | 95.4 | 95.0 |  | 94.8 | 94.6 | 94.7 | 94.7 | 95.2 | 95.1 |
|  |  | 5% | 95.4 | 94.9 | 95.2 | 95.1 | 95.1 | 95.2 |  | 94.7 | 94.8 | 95.0 | 95.2 | 95.4 | 95.4 |
| Type I error | $\pi$ | 50% | 4.7 | 71.1 | 99.2 | 100.0 | 100.0 | 100.0 |  | 14.5 | 89.4 | 99.8 | 100.0 | 100.0 | 100.0 |
| and power |  | 10% | 4.3 | 38.4 | 81.4 | 96.5 | 100.0 | 100.0 |  | 9.5 | 54.9 | 89.0 | 98.1 | 100.0 | 100.0 |
| (%) |  | 5% | 4.5 | 24.0 | 56.5 | 80.1 | 99.9 | 100.0 |  | 7.6 | 34.7 | 66.8 | 85.9 | 99.9 | 100.0 |
|  | $\pi^{*}$ | 50% | 4.6 | 70.4 | 99.0 | 100.0 | 100.0 | 100.0 |  | 5.4 | 79.0 | 99.5 | 100.0 | 100.0 | 100.0 |
|  |  | 10% | 4.9 | 37.3 | 80.1 | 96.1 | 100.0 | 100.0 |  | 5.2 | 43.7 | 84.7 | 97.3 | 100.0 | 100.0 |
|  |  | 5% | 4.5 | 23.6 | 55.0 | 78.8 | 99.8 | 100.0 |  | 5.3 | 27.6 | 60.0 | 82.7 | 99.8 | 100.0 |

[Note] Scenario I: the situation wherein the covariate distribution is similar between the randomized control trial (RCT) data and historical control data; Scenario II: the situation wherein the covariate distribution is not similar between the RCT data and historical control data; $\pi$ (without $X_{r}$): the conventional method; $\pi^{*}$ (with $X_{r}$): the proposed method.

Performance of the estimated propensity score (PS) model by simulation setting assuming the randomized assignment of treatment variables and $n=200$

|  |  |  | **Scenario Ⅰ** | | | | | |  | **Scenario Ⅱ** | | | | | |
| --- | --- | --- | --- | --- | --- | --- | --- | --- | --- | --- | --- | --- | --- | --- | --- |
|  |  |  | Odds ratio | | | | | |  | Odds ratio | | | | | |
| Performance measurement | PS model | Outcome event rate | 1.0 | 1.5 | 2.0 | 2.5 | 5.0 | 10.0 |  | 1.0 | 1.5 | 2.0 | 2.5 | 5.0 | 10.0 |
| Bias | $\pi$ | 50% | -0.001 | -0.009 | -0.014 | -0.018 | -0.014 | 0.014 |  | 0.152 | 0.145 | 0.140 | 0.142 | 0.155 | 0.191 |
|  | (without $X_{r}$) | 10% | -0.170 | -0.060 | -0.039 | -0.030 | 0.006 | 0.074 |  | -0.009 | 0.074 | 0.103 | 0.120 | 0.155 | 0.248 |
|  |  | 5% | -1.392 | -0.644 | -0.325 | -0.140 | 0.273 | 1.168 |  | -1.174 | -0.463 | -0.153 | 0.001 | 0.479 | 1.410 |
|  | $\pi^{*}$ | 50% | 0.000 | -0.009 | -0.014 | -0.019 | -0.015 | 0.013 |  | 0.041 | 0.032 | 0.026 | 0.027 | 0.032 | 0.058 |
|  | (with $X_{r}$) | 10% | -0.156 | -0.051 | -0.031 | -0.023 | 0.016 | 0.090 |  | -0.086 | -0.015 | 0.009 | 0.024 | 0.056 | 0.151 |
|  |  | 5% | -1.333 | -0.608 | -0.296 | -0.114 | 0.314 | 1.242 |  | -1.182 | -0.504 | -0.205 | -0.057 | 0.420 | 1.376 |
| MSE | $\pi$ | 50% | 0.117 | 0.118 | 0.120 | 0.127 | 0.164 | 0.370 |  | 0.163 | 0.164 | 0.169 | 0.176 | 0.257 | 0.499 |
|  |  | 10% | 2.334 | 0.642 | 0.479 | 0.395 | 0.313 | 0.961 |  | 1.850 | 0.824 | 0.583 | 0.423 | 0.356 | 1.316 |
|  |  | 5% | 25.968 | 12.404 | 7.314 | 4.893 | 6.312 | 20.210 |  | 23.863 | 11.404 | 6.920 | 5.209 | 7.141 | 22.227 |
|  | $\pi^{*}$ | 50% | 0.119 | 0.120 | 0.124 | 0.131 | 0.166 | 0.360 |  | 0.121 | 0.119 | 0.125 | 0.131 | 0.200 | 0.393 |
|  |  | 10% | 2.145 | 0.620 | 0.471 | 0.394 | 0.340 | 1.047 |  | 1.635 | 0.714 | 0.499 | 0.362 | 0.315 | 1.306 |
|  |  | 5% | 24.422 | 11.771 | 7.020 | 4.781 | 6.745 | 21.589 |  | 22.407 | 10.712 | 6.575 | 5.024 | 7.492 | 23.324 |
| Coverage | $\pi$ | 50% | 95.2 | 95.4 | 95.6 | 95.3 | 94.9 | 95.1 |  | 93.1 | 93.5 | 93.6 | 94.0 | 94.0 | 94.2 |
| (%) |  | 10% | 95.8 | 96.3 | 96.1 | 96.2 | 95.8 | 96.3 |  | 93.1 | 93.3 | 94.0 | 94.1 | 94.4 | 95.5 |
|  |  | 5% | 89.5 | 94.0 | 95.5 | 96.2 | 95.6 | 90.2 |  | 87.8 | 91.9 | 92.7 | 93.8 | 94.4 | 89.9 |
|  | $\pi^{*}$ | 50% | 95.1 | 95.4 | 95.6 | 95.2 | 95.1 | 95.4 |  | 95.3 | 95.7 | 95.6 | 95.7 | 95.4 | 96.0 |
|  |  | 10% | 95.7 | 96.0 | 95.8 | 95.9 | 95.3 | 95.4 |  | 95.6 | 95.7 | 95.7 | 95.8 | 95.2 | 95.3 |
|  |  | 5% | 89.2 | 93.4 | 95.0 | 95.8 | 95.0 | 90.3 |  | 89.4 | 93.5 | 94.7 | 95.3 | 94.8 | 89.8 |
| Type I error | $\pi$ | 50% | 4.7 | 20.2 | 48.8 | 72.4 | 99.1 | 100.0 |  | 6.6 | 31.2 | 59.5 | 78.6 | 98.9 | 99.9 |
| and power |  | 10% | 4.1 | 12.0 | 27.6 | 43.4 | 88.5 | 99.1 |  | 6.8 | 18.0 | 34.8 | 50.4 | 89.5 | 99.0 |
| (%) |  | 5% | 10.5 | 12.1 | 19.1 | 27.8 | 65.3 | 89.2 |  | 12.0 | 15.2 | 23.6 | 32.7 | 68.0 | 89.6 |
|  | $\pi^{*}$ | 50% | 4.7 | 19.6 | 48.0 | 71.0 | 99.0 | 100.0 |  | 4.6 | 22.8 | 52.3 | 74.9 | 99.4 | 100.0 |
|  |  | 10% | 4.1 | 12.0 | 27.1 | 42.2 | 86.8 | 98.9 |  | 4.2 | 14.0 | 29.8 | 45.6 | 88.5 | 99.0 |
|  |  | 5% | 10.7 | 12.4 | 19.3 | 27.8 | 63.5 | 87.5 |  | 10.5 | 12.7 | 20.6 | 29.9 | 65.2 | 88.3 |

[Note] Scenario I: the situation wherein the covariate distribution is similar between the randomized control trial (RCT) data and historical control data; Scenario II: the situation wherein the covariate distribution is not similar between the RCT data and historical control data; $\pi$ (without $X_{r}$): the conventional method; $\pi^{*}$ (with $X_{r}$): the proposed method.

1. **Probability of treatment allocation correction value** $\boldsymbol{b}_{\boldsymbol{0}}$ **and outcome event rate correction value** $\boldsymbol{a}_{\boldsymbol{0}}$

The probability of the treatment allocation correction value $b_{0}$ in the true propensity score model and outcome event rate correction value $a_{0}$ in the model that generates outcome data are shown below.

Probability of treatment allocation correction value $b_{0}$ in the true propensity score model

| **Scenario Ⅰ** | **Scenario Ⅱ** |
| --- | --- |
| -1.37 | 1.16 |

Outcome event rate correction value $a_{0}$ in the model that generates outcome data

| **Scenario Ⅰ** | | | | | | |
| --- | --- | --- | --- | --- | --- | --- |
| Outcome  event rate | Odds ratio | | | | | |
|  | 1.0 | 1.5 | 2.0 | 2.5 | 5.0 | 10.0 |
| 50% | 0.000 | -0.100 | -0.170 | -0.222 | -0.368 | -0.484 |
| 10% | -2.249 | -2.371 | -2.474 | -2.564 | -2.897 | -3.314 |
| 5% | -3.003 | -3.128 | -3.237 | -3.333 | -3.698 | -4.161 |
| **Scenario Ⅱ** | | | | | | |
| Outcome  event rate | Odds ratio | | | | | |
|  | 1.0 | 1.5 | 2.0 | 2.5 | 5.0 | 10.0 |
| 50% | 0.094 | -0.006 | -0.076 | -0.127 | -0.272 | -0.386 |
| 10% | -2.155 | -2.279 | -2.383 | -2.474 | -2.810 | -3.229 |
| 5% | -2.909 | -3.036 | -3.146 | -3.243 | -3.610 | -4.076 |

The above correction values were used in the simulation setting that assumes the allocation ratio between the RCT treatment group, RCT control group, and historical control group as 1:1:2, as in the main text. Correction values $b_{0}$ and $a_{0}$ used in the simulation setting assuming the other allocation ratios (Appendix Table B.1 and Appendix Table B.2) are shown below.

Probability of treatment allocation correction value $b_{0}$ in the true propensity score model in the simulation setting assuming the other allocation ratios

| **Rt: Rc: H** | **Scenario Ⅰ** | **Scenario Ⅱ** |
| --- | --- | --- |
| 2：1：3 | -0.87 | 2.02 |
| 9：1：10 | -0.25 | 3.79 |
| 1：1：4 | -1.99 | 1.16 |
| 2：1：6 | -1.56 | 2.02 |
| 9：1：20 | -1.07 | 3.79 |
| 1：1：18 | -3.5 | 1.16 |
| 2：1：27 | -3.17 | 2.02 |
| 9：1：90 | -2.8 | 3.79 |
| 2：1：1 | 0.00 | 2.02 |
| 3：1：2 | 0.00 | 2.51 |

[Note] R: RCT, H: Historical control, t: treatment group, c: control group.

Outcome event rate correction value $a_{0}$ in the model that generates outcome data in the simulation setting, assuming Rt:Rc:Hc = 2:1:3

| **Scenario Ⅰ** | | | | | | |
| --- | --- | --- | --- | --- | --- | --- |
| Outcome  event rate | Odds ratio | | | | | |
|  | 1.0 | 1.5 | 2.0 | 2.5 | 5.0 | 10.0 |
| 50% | 0.000 | -0.135 | -0.229 | -0.300 | -0.509 | -0.689 |
| 10% | -2.249 | -2.409 | -2.540 | -2.653 | -3.061 | -3.554 |
| 5% | -3.003 | -3.166 | -3.303 | -3.422 | -3.858 | -4.383 |
| **Scenario Ⅱ** | | | | | | |
| Outcome  event rate | Odds ratio | | | | | |
|  | 1.0 | 1.5 | 2.0 | 2.5 | 5.0 | 10.0 |
| 50% | 0.094 | -0.041 | -0.135 | -0.206 | -0.414 | -0.594 |
| 10% | -2.155 | -2.315 | -2.447 | -2.560 | -2.970 | -3.464 |
| 5% | -2.909 | -3.073 | -3.210 | -3.329 | -3.765 | -4.292 |

[Note] R: RCT, H: historical control, t: treatment group, c: control group.

Outcome event rate correction value $a_{0}$ in the model that generates outcome data in the simulation setting assuming Rt:Rc:Hc = 9:1:10

| **Scenario Ⅰ** | | | | | | |
| --- | --- | --- | --- | --- | --- | --- |
| Outcome  event rate | Odds ratio | | | | | |
|  | 1.0 | 1.5 | 2.0 | 2.5 | 5.0 | 10.0 |
| 50% | 0.000 | -0.183 | -0.311 | -0.411 | -0.716 | -1.010 |
| 10% | -2.250 | -2.458 | -2.625 | -2.766 | -3.258 | -3.823 |
| 5% | -3.004 | -3.216 | -3.388 | -3.534 | -4.046 | -4.629 |
| **Scenario Ⅱ** | | | | | | |
| Outcome  event rate | Odds ratio | | | | | |
|  | 1.0 | 1.5 | 2.0 | 2.5 | 5.0 | 10.0 |
| 50% | 0.094 | -0.089 | -0.217 | -0.317 | -0.621 | -0.915 |
| 10% | -2.155 | -2.362 | -2.528 | -2.667 | -3.157 | -3.719 |
| 5% | -2.909 | -3.119 | -3.290 | -3.434 | -3.943 | -4.525 |

[Note] R: RCT, H: historical control, t: treatment group, c: control group.

Outcome event rate correction value $a_{0}$ in the model that generates outcome data in the simulation setting assuming Rt:Rc:Hc = 1:1:4

| **Scenario Ⅰ** | | | | | | |
| --- | --- | --- | --- | --- | --- | --- |
| Outcome  event rate | Odds ratio | | | | | |
|  | 1.0 | 1.5 | 2.0 | 2.5 | 5.0 | 10.0 |
| 50% | 0.000 | -0.067 | -0.112 | -0.145 | -0.234 | -0.300 |
| 10% | -2.249 | -2.332 | -2.403 | -2.466 | -2.706 | -3.015 |
| 5% | -3.003 | -3.089 | -3.165 | -3.234 | -3.506 | -3.873 |
| **Scenario Ⅱ** | | | | | | |
| Outcome  event rate | Odds ratio | | | | | |
|  | 1.0 | 1.5 | 2.0 | 2.5 | 5.0 | 10.0 |
| 50% | -2.141 | -2.225 | -2.298 | -2.362 | -2.604 | -2.914 |
| 10% | -2.895 | -2.982 | -3.059 | -3.129 | -3.405 | -3.775 |
| 5% | 0.108 | 0.042 | -0.003 | -0.036 | -0.125 | -0.190 |

[Note] R: RCT, H: historical control, t: treatment group, c: control group.

Outcome event rate correction value $a_{0}$ in the model that generates outcome data in the simulation setting assuming Rt:Rc:Hc = 2:1:6

| **Scenario Ⅰ** | | | | | | |
| --- | --- | --- | --- | --- | --- | --- |
| Outcome  event rate | Odds ratio | | | | | |
|  | 1.0 | 1.5 | 2.0 | 2.5 | 5.0 | 10.0 |
| 50% | 0.000 | -0.089 | -0.150 | -0.196 | -0.322 | -0.420 |
| 10% | -2.249 | -2.358 | -2.451 | -2.532 | -2.836 | -3.220 |
| 5% | -3.003 | -3.116 | -3.214 | -3.301 | -3.637 | -4.073 |
| **Scenario Ⅱ** | | | | | | |
| Outcome  event rate | Odds ratio | | | | | |
|  | 1.0 | 1.5 | 2.0 | 2.5 | 5.0 | 10.0 |
| 50% | 0.108 | 0.019 | -0.042 | -0.087 | -0.213 | -0.311 |
| 10% | -2.141 | -2.251 | -2.344 | -2.425 | -2.730 | -3.115 |
| 5% | -2.895 | -3.007 | -3.106 | -3.193 | -3.530 | -3.966 |

[Note] R: RCT, H: historical control, t: treatment group, c: control group.

Outcome event rate correction value $a_{0}$ in the model that generates outcome data in the simulation setting assuming Rt:Rc:Hc = 9:1:20

| **Scenario Ⅰ** | | | | | | |
| --- | --- | --- | --- | --- | --- | --- |
| Outcome  event rate | Odds ratio | | | | | |
|  | 1.0 | 1.5 | 2.0 | 2.5 | 5.0 | 10.0 |
| 50% | 0.000 | -0.120 | -0.204 | -0.267 | -0.448 | -0.600 |
| 10% | -2.248 | -2.393 | -2.512 | -2.616 | -2.995 | -3.460 |
| 5% | -3.002 | -3.150 | -3.276 | -3.385 | -3.794 | -4.297 |
| **Scenario Ⅱ** | | | | | | |
| Outcome  event rate | Odds ratio | | | | | |
|  | 1.0 | 1.5 | 2.0 | 2.5 | 5.0 | 10.0 |
| 50% | 0.108 | -0.012 | -0.096 | -0.160 | -0.343 | -0.497 |
| 10% | -2.141 | -2.284 | -2.403 | -2.505 | -2.882 | -3.344 |
| 5% | -2.895 | -3.040 | -3.165 | -3.273 | -3.678 | -4.178 |

[Note] R: RCT, H: historical control, t: treatment group, c: control group.

Outcome event rate correction value $a_{0}$ in the model that generates outcome data in the simulation setting assuming Rt:Rc:Hc = 1:1:18

| **Scenario Ⅰ** | | | | | | |
| --- | --- | --- | --- | --- | --- | --- |
| Outcome  event rate | Odds ratio | | | | | |
|  | 1.0 | 1.5 | 2.0 | 2.5 | 5.0 | 10.0 |
| 50% | 0.000 | -0.020 | -0.033 | -0.042 | -0.066 | -0.082 |
| 10% | -2.249 | -2.275 | -2.298 | -2.319 | -2.397 | -2.494 |
| 5% | -3.004 | -3.031 | -3.056 | -3.079 | -3.176 | -3.316 |
| **Scenario Ⅱ** | | | | | | |
| Outcome  event rate | Odds ratio | | | | | |
|  | 1.0 | 1.5 | 2.0 | 2.5 | 5.0 | 10.0 |
| 50% | 0.128 | 0.109 | 0.096 | 0.086 | 0.063 | 0.047 |
| 10% | -2.121 | -2.147 | -2.170 | -2.191 | -2.270 | -2.368 |
| 5% | -2.875 | -2.902 | -2.927 | -2.950 | -3.049 | -3.190 |

[Note] R: RCT, H: historical control, t: treatment group, c: control group.

Outcome event rate correction value $a_{0}$ in the model that generates outcome data in the simulation setting assuming Rt:Rc:Hc = 2:1:27

| **Scenario Ⅰ** | | | | | | |
| --- | --- | --- | --- | --- | --- | --- |
| Outcome  event rate | Odds ratio | | | | | |
|  | 1.0 | 1.5 | 2.0 | 2.5 | 5.0 | 10.0 |
| 50% | 0.000 | -0.026 | -0.044 | -0.056 | -0.088 | -0.110 |
| 10% | -2.249 | -2.284 | -2.314 | -2.341 | -2.444 | -2.573 |
| 5% | -3.003 | -3.039 | -3.072 | -3.102 | -3.228 | -3.408 |
| **Scenario Ⅱ** | | | | | | |
| Outcome  event rate | Odds ratio | | | | | |
|  | 1.0 | 1.5 | 2.0 | 2.5 | 5.0 | 10.0 |
| 50% | 0.128 | 0.102 | 0.084 | 0.072 | 0.039 | 0.018 |
| 10% | -2.121 | -2.155 | -2.185 | -2.212 | -2.316 | -2.446 |
| 5% | -2.875 | -2.910 | -2.943 | -2.973 | -3.099 | -3.280 |

[Note] R: RCT, H: historical control, t: treatment group, c: control group.

Outcome event rate correction value $a_{0}$ in the model that generates outcome data in the simulation setting assuming Rt:Rc:Hc = 9:1:90

| **Scenario Ⅰ** | | | | | | |
| --- | --- | --- | --- | --- | --- | --- |
| Outcome  event rate | Odds ratio | | | | | |
|  | 1.0 | 1.5 | 2.0 | 2.5 | 5.0 | 10.0 |
| 50% | 0.000 | -0.036 | -0.060 | -0.077 | -0.122 | -0.152 |
| 10% | -2.249 | -2.295 | -2.335 | -2.371 | -2.510 | -2.686 |
| 5% | -3.003 | -3.051 | -3.095 | -3.135 | -3.300 | -3.535 |
| **Scenario Ⅱ** | | | | | | |
| Outcome  event rate | Odds ratio | | | | | |
|  | 1.0 | 1.5 | 2.0 | 2.5 | 5.0 | 10.0 |
| 50% | 0.128 | 0.093 | 0.068 | 0.051 | 0.006 | -0.026 |
| 10% | -2.121 | -2.165 | -2.205 | -2.240 | -2.376 | -2.550 |
| 5% | -2.875 | -2.921 | -2.963 | -3.002 | -3.164 | -3.394 |

[Note] R: RCT, H: historical control, t: treatment group, c: control group.

Outcome event rate correction value $a_{0}$ in the model that generates outcome data in the simulation setting assuming Rt:Rc:Hc = 1:1:2

| **Scenario Ⅰ** | | | | | | |
| --- | --- | --- | --- | --- | --- | --- |
| Outcome  event rate | Odds ratio | | | | | |
|  | 1.0 | 1.5 | 2.0 | 2.5 | 5.0 | 10.0 |
| 50% | 0 | -0.203 | -0.347 | -0.458 | -0.805 | -1.151 |
| 10% | -2.249 | -2.478 | -2.659 | -2.81 | -3.331 | -3.918 |
| 5% | -3.004 | -3.235 | -3.421 | -3.577 | -4.115 | -4.717 |
| **Scenario Ⅱ** | | | | | | |
| Outcome  event rate | Odds ratio | | | | | |
|  | 1.0 | 1.5 | 2.0 | 2.5 | 5.0 | 10.0 |
| 50% | 0.072 | -0.131 | -0.275 | -0.387 | -0.734 | -1.082 |
| 10% | -2.176 | -2.406 | -2.588 | -2.739 | -3.262 | -3.85 |
| 5% | -2.93 | -3.164 | -3.351 | -3.507 | -4.046 | -4.649 |

[Note] R: RCT, H: historical control, t: treatment group, c: control group.

Outcome event rate correction value $a_{0}$ in the model that generates outcome data in the simulation setting assuming Rt:Rc:Hc = 3:1:2

| **Scenario Ⅰ** | | | | | | |
| --- | --- | --- | --- | --- | --- | --- |
| Outcome  event rate | Odds ratio | | | | | |
|  | 1.0 | 1.5 | 2.0 | 2.5 | 5.0 | 10.0 |
| 50% | 0 | -0.203 | -0.347 | -0.458 | -0.805 | -1.151 |
| 10% | -2.249 | -2.478 | -2.659 | -2.81 | -3.331 | -3.918 |
| 5% | -3.004 | -3.235 | -3.421 | -3.577 | -4.115 | -4.717 |
| **Scenario Ⅱ** | | | | | | |
| Outcome  event rate | Odds ratio | | | | | |
|  | 1.0 | 1.5 | 2.0 | 2.5 | 5.0 | 10.0 |
| 50% | 0.079 | -0.123 | -0.267 | -0.379 | -0.726 | -1.073 |
| 10% | -2.17 | -2.398 | -2.58 | -2.73 | -3.252 | -3.839 |
| 5% | -2.923 | -3.156 | -3.342 | -3.497 | -4.035 | -4.638 |

[Note] R: RCT, H: historical control, t: treatment group, c: control group.

In addition, correction values $b_{0}$ and $a_{0}$ used in the simulation setting assuming that one of the covariates is binary data (Appendix C) are shown below.

Probability of treatment allocation correction value $b_{0}$ in the true propensity score model in the simulation setting assuming three continuous covariates and one binary covariate

| **Rt: Rc: H** | **Scenario Ⅰ** | **Scenario Ⅱ** |
| --- | --- | --- |
| 1：1：2 | -0.05 | 1.11 |

[Note] R: RCT, H: Historical control, t: treatment group, c: control group.

Outcome event rate correction value $a_{0}$ in the model that generates outcome data in the simulation setting assuming three continuous covariates and one binary covariate

| **Scenario Ⅰ** | | | | | | |
| --- | --- | --- | --- | --- | --- | --- |
| Outcome  event rate | Odds ratio | | | | | |
|  | 1.0 | 1.5 | 2.0 | 2.5 | 5.0 | 10.0 |
| 50% | 0.069 | -0.134 | -0.278 | -0.390 | -0.736 | -1.083 |
| 10% | -2.175 | -2.404 | -2.586 | -2.737 | -3.259 | -3.847 |
| 5% | -2.928 | -3.160 | -3.347 | -3.502 | -4.041 | -4.645 |
| **Scenario Ⅱ** | | | | | | |
| Outcome  event rate | Odds ratio | | | | | |
|  | 1.0 | 1.5 | 2.0 | 2.5 | 5.0 | 10.0 |
| 50% | 0.162 | 0.061 | -0.007 | -0.059 | -0.203 | -0.317 |
| 10% | -2.082 | -2.206 | -2.310 | -2.400 | -2.738 | -3.159 |
| 5% | -2.835 | -2.963 | -3.073 | -3.170 | -3.539 | -4.006 |

[Note] R: RCT, H: historical control, t: treatment group, c: control group.

Then, correction values $b_{0}$ and $a_{0}$ used in the simulation setting that assumes the randomized assignment of treatment variables (Appendix D) are shown below.

Probability of treatment allocation correction value $b_{0}$ in the true propensity score model in the simulation setting assuming the randomized assignment of treatment variables

| **Rt: Rc: H** | **Scenario Ⅰ** | **Scenario Ⅱ** |
| --- | --- | --- |
| 1：1：2 | -0.87 | 2.02 |

[Note] R: RCT, H: Historical control, t: treatment group, c: control group.

Outcome event rate correction value $a_{0}$ in the model that generates outcome data in the simulation setting assuming the randomized assignment of treatment variables

| **Scenario Ⅰ** | | | | | | |
| --- | --- | --- | --- | --- | --- | --- |
| Outcome  event rate | Odds ratio | | | | | |
|  | 1.0 | 1.5 | 2.0 | 2.5 | 5.0 | 10.0 |
| 50% | 0.000 | -0.101 | -0.171 | -0.223 | -0.374 | -0.495 |
| 10% | -2.248 | -2.362 | -2.459 | -2.543 | -2.861 | -3.261 |
| 5% | -3.002 | -3.118 | -3.219 | -3.309 | -3.654 | -4.099 |
| **Scenario Ⅱ** | | | | | | |
| Outcome  event rate | Odds ratio | | | | | |
|  | 1.0 | 1.5 | 2.0 | 2.5 | 5.0 | 10.0 |
| 50% | 0.094 | -0.007 | -0.076 | -0.129 | -0.277 | -0.396 |
| 10% | -2.155 | -2.272 | -2.371 | -2.458 | -2.782 | -3.191 |
| 5% | -2.909 | -3.029 | -3.133 | -3.225 | -3.579 | -4.031 |

[Note] R: RCT, H: historical control, t: treatment group, c: control group.

1. **Calculation method of the true PS model in scenario (II)**

The calculation method of the true PS model $\pi_{i,true}^{*}$ in the situation wherein the distribution of covariates is not similar between the RCT data and historical control data is shown below. First, the true PS model only for RCT data,

$$\begin{aligned} \pi_{i,true,RCT}=\mathrm{logit}\left\{ \Pr\left( T=1 | X \right) \right\}=b_{0}+{1.00X}_{i1}-{0.50X}_{i2}+{0.25X}_{i3}+{0.10X}_{i4},\#\left( F.1 \right) \end{aligned}$$

and the true propensity score model for only the historical control data,

$$\begin{aligned} \pi_{i,true,HC}=\mathrm{logit}\left\{ \Pr\left( T=1 \right) \right\}=0,\#\left( F.2 \right) \end{aligned}$$

were used to give each participant a pretreatment allocation $T_{i,pre}$. At this time, using a Bernoulli distribution based on the probability ($\pi_{i,true,RCT}$ or $\pi_{i,true,HC}$) given by the above equation,

$$\begin{aligned} T_{i,pre} \sim Bernoulli\left\{ \exp\mathrm{it}\left( \pi_{i,true,RCT or HC} \right) \right\},\#\left( F.3 \right) \end{aligned}$$

RCT data were given $T_{i,pre}=1 or 0$ and historical control data were all given $T_{i,pre}=0$.

Next, the PS model was estimated using a logistic regression model with pretreatment allocation $T_{i,pre}$ as the objective variable; covariates $X_{i1},X_{i2},X_{i3}, andX_{i4}$; and variable $X_{ir}$ whether the data are RCT data or not were used as explanatory variables.

Finally, this was repeated 10,000 times, and the average value of the coefficient for each variable was determined as the coefficient of the true PS model in scenario (II).

In addition, coefficient value $b_{r}$ of index variable $X_{r}$ for the information on whether the data are RCT data or not in the true propensity score model is shown below.

Coefficient value $b_{r}$ of index variable $X_{r}$ for the information on whether the data are randomized controlled trial data or not in the true propensity score model

| **Rt: Rc: H** | **Scenario Ⅱ** |
| --- | --- |
| 1：1：2 | 11.11 |
| 2：1：3 | 11.55 |
| 9：1：10 | 12.44 |
| 1：1：4 | 11.13 |
| 2：1：6 | 11.56 |
| 9：1：20 | 12.95 |
| 1：1：18 | 12.14 |
| 2：1：27 | 12.58 |
| 9：1：90 | 13.75 |
| 2：1：1 | 11.02 |
| 3：1：2 | 11.28 |

[Note] R: RCT, H: Historical control, t: treatment group, c: control group.

Similarly, in the case, where one of the covariates is binary data (Appendix C) or that the randomized assignment of treatment variables (Appendix D), coefficient value $b_{r}$ of index variable $X_{r}$ for the information on whether the data are RCT data or not in the true propensity score model in the case of that are shown below.

Coefficient value $b_{r}$ of index variable $X_{r}$ for the information on whether the data are randomized controlled trial data or not in the true propensity score model in the simulation setting three continuous covariates and one binary covariate

| **Rt: Rc: H** | **Scenario Ⅱ** |
| --- | --- |
| 1：1：2 | 11.11 |

[Note] R: RCT, H: Historical control, t: treatment group, c: control group.

Coefficient value $b_{r}$ of index variable $X_{r}$ for the information on whether the data are randomized controlled trial data or not in the true propensity score model in the simulation setting assuming the randomized assignment of treatment variables

| **Rt: Rc: H** | **Scenario Ⅱ** |
| --- | --- |
| 1：1：2 | 11.11 |

[Note] R: RCT, H: Historical control, t: treatment group, c: control group.

1. **Simulation based on actual clinical trial parameter settings**

The text presented the simulation result by setting that four covariates are generated from the multivariate standard normal distribution like Equation (7) or Equation (8). And, in scenario (II), after generating covariates, each covariate in the RCT data are transformed as Equation (9).

In this chapter, the simulations refer to parameter settings from actual clinical trials was also examined. The clinical trial used for reference is the “BREEZE-AD PEDS phase III” trial [29]. The methods and conditions for the simulation settings are the same as those shown in the text, except the covariates are generated based on parameters referenced to clinical trial, unless otherwise noted. We set the total number of participants as $n=480$, the reason is that each group in the RCT was 120 participants based on this clinical trial, and the allocation ratio between the RCT treatment group, RCT control group, and historical control group as 1:1:2. The method of assignment treatment variables is randomized (Appendix D).

With reference to the “BREEZE-AD PEDS phase III” trial, continuous and binary covariates were generated for participant $i$ as

$$\begin{aligned} \left\{ X_{i1}\mathrm{or}{X^{'}}_{i1} \right\}\sim N\left( 12.0, {3.9}^{2} \right),\#\left( G.1 \right) \end{aligned}$$

$$\begin{aligned} \left\{ X_{i2}\mathrm{or}{X^{'}}_{i2} \right\}\sim Bernoulli\left( 0.38 \right),\#\left( G.2 \right) \end{aligned}$$

$$\begin{aligned} \left\{ X_{i3}\mathrm{or}{X^{'}}_{i3} \right\}\sim N\left( 2.7, {3.8}^{2} \right),\#\left( G.3 \right) \end{aligned}$$

$$\begin{aligned} \left\{ X_{i4}\mathrm{or}{X^{'}}_{i4} \right\}\sim Bernoulli\left( 0.50 \right).\#\left( G.4 \right) \end{aligned}$$

In scenario (II), after generating covariates, each covariate in the historical control data are transformed as follows:

$$\begin{aligned} \left\{ X_{i1}={X^{'}}_{i1}+1, X_{i2}={X^{'}}_{i2}\div0.7, X_{i3}={X^{'}}_{i3},X_{i4}={X^{'}}_{i4} \right\}.\#\left( G.5 \right) \end{aligned}$$

For RCT data, the covariates are without transformation, $X_{i1},X_{i2},X_{i3},{and X}_{i4}$ generated based on the clinical trial setting is simply used, that is,

$$\begin{aligned} \left\{ X_{i1}={X^{'}}_{i1}, X_{i2}={X^{'}}_{i2}, X_{i3}={X^{'}}_{i3},X_{i4}={X^{'}}_{i4} \right\}.\#\left( G.6 \right) \end{aligned}$$

The simulation results using covariates generated with these settings are shown below.

Performance of the estimated propensity score (PS) model by simulation based on parameter settings from actual clinical trial

|  |  |  | **Scenario Ⅰ** | | | | | |  | **Scenario Ⅱ** | | | | | |
| --- | --- | --- | --- | --- | --- | --- | --- | --- | --- | --- | --- | --- | --- | --- | --- |
|  |  |  | Odds ratio | | | | | |  | Odds ratio | | | | | |
| Performance measurement | PS model | Outcome event rate | 1.0 | 1.5 | 2.0 | 2.5 | 5.0 | 10.0 |  | 1.0 | 1.5 | 2.0 | 2.5 | 5.0 | 10.0 |
| Bias | $\pi$ | 50% | 0.000 | -0.027 | -0.046 | -0.060 | -0.102 | -0.138 |  | 0.158 | 0.130 | 0.112 | 0.096 | 0.055 | 0.021 |
|  | (without $X_{r}$) | 10% | -0.028 | -0.034 | -0.037 | -0.042 | -0.058 | -0.067 |  | 0.132 | 0.125 | 0.121 | 0.115 | 0.100 | 0.090 |
|  |  | 5% | -0.098 | -0.053 | -0.036 | -0.027 | -0.011 | 0.042 |  | 0.051 | 0.103 | 0.122 | 0.132 | 0.149 | 0.200 |
|  | $\pi^{*}$ | 50% | 0.000 | -0.027 | -0.046 | -0.060 | -0.102 | -0.138 |  | -0.010 | -0.038 | -0.056 | -0.071 | -0.113 | -0.149 |
|  | (with $X_{r}$) | 10% | -0.026 | -0.032 | -0.036 | -0.041 | -0.055 | -0.062 |  | -0.038 | -0.045 | -0.048 | -0.053 | -0.067 | -0.075 |
|  |  | 5% | -0.093 | -0.049 | -0.031 | -0.022 | -0.004 | 0.054 |  | -0.114 | -0.064 | -0.044 | -0.035 | -0.016 | 0.041 |
| MSE | $\pi$ | 50% | 0.041 | 0.044 | 0.047 | 0.050 | 0.067 | 0.094 |  | 0.069 | 0.063 | 0.060 | 0.058 | 0.063 | 0.078 |
|  |  | 10% | 0.136 | 0.117 | 0.108 | 0.104 | 0.107 | 0.126 |  | 0.162 | 0.140 | 0.128 | 0.121 | 0.117 | 0.132 |
|  |  | 5% | 1.038 | 0.443 | 0.319 | 0.240 | 0.216 | 0.736 |  | 1.194 | 0.506 | 0.349 | 0.269 | 0.245 | 0.805 |
|  | $\pi^{*}$ | 50% | 0.044 | 0.046 | 0.049 | 0.052 | 0.069 | 0.095 |  | 0.044 | 0.046 | 0.050 | 0.053 | 0.071 | 0.098 |
|  |  | 10% | 0.139 | 0.121 | 0.113 | 0.109 | 0.114 | 0.137 |  | 0.141 | 0.123 | 0.114 | 0.110 | 0.115 | 0.137 |
|  |  | 5% | 1.016 | 0.442 | 0.323 | 0.250 | 0.234 | 0.792 |  | 1.145 | 0.475 | 0.326 | 0.252 | 0.233 | 0.818 |
| Coverage | $\pi$ | 50% | 96.0 | 95.6 | 95.3 | 94.9 | 93.1 | 91.5 |  | 90.0 | 91.9 | 93.2 | 94.1 | 95.3 | 96.1 |
| (%) |  | 10% | 95.6 | 95.9 | 95.7 | 95.5 | 95.1 | 94.4 |  | 92.7 | 93.2 | 93.6 | 94.1 | 94.8 | 95.5 |
|  |  | 5% | 96.4 | 96.3 | 96.2 | 96.0 | 95.6 | 95.9 |  | 93.2 | 94.0 | 94.2 | 94.5 | 95.2 | 96.5 |
|  | $\pi^{*}$ | 50% | 95.7 | 95.5 | 95.1 | 95.0 | 92.8 | 91.4 |  | 95.7 | 95.4 | 94.8 | 94.4 | 92.3 | 90.9 |
|  |  | 10% | 95.6 | 95.6 | 95.4 | 95.4 | 95.0 | 94.2 |  | 95.7 | 95.4 | 95.3 | 95.4 | 94.8 | 94.0 |
|  |  | 5% | 96.2 | 96.2 | 96.1 | 95.8 | 95.6 | 95.0 |  | 96.3 | 96.3 | 96.0 | 95.8 | 95.6 | 94.9 |
| Type I error | $\pi$ | 50% | 3.9 | 41.0 | 85.5 | 97.9 | 100.0 | 100.0 |  | 10.0 | 68.6 | 95.5 | 99.6 | 100.0 | 100.0 |
| and power |  | 10% | 4.3 | 21.6 | 53.4 | 77.3 | 99.8 | 100.0 |  | 7.2 | 36.2 | 68.2 | 86.9 | 99.9 | 100.0 |
| (%) |  | 5% | 3.6 | 14.6 | 34.3 | 54.0 | 94.6 | 99.8 |  | 6.8 | 23.4 | 46.3 | 65.5 | 96.8 | 99.9 |
|  | $\pi^{*}$ | 50% | 4.3 | 39.7 | 84.3 | 97.6 | 100.0 | 100.0 |  | 4.2 | 37.7 | 83.1 | 97.3 | 100.0 | 100.0 |
|  |  | 10% | 4.3 | 21.0 | 52.0 | 76.1 | 99.7 | 100.0 |  | 4.3 | 20.2 | 50.6 | 74.8 | 99.7 | 100.0 |
|  |  | 5% | 3.7 | 14.2 | 33.1 | 52.8 | 93.8 | 99.8 |  | 3.6 | 13.7 | 32.3 | 51.9 | 93.6 | 99.8 |

[Note] Scenario I: the situation wherein the covariate distribution is similar between the randomized control trial (RCT) data and historical control data; Scenario II: the situation wherein the covariate distribution is not similar between the RCT data and historical control data; $\pi$ (without $X_{r}$): the conventional method; $\pi^{*}$ (with $X_{r}$): the proposed method.

The baseline patient demographics and disease characteristics information from the actual clinical trial that was used as reference for covariates parameters is shown below.

Baseline patient demographics and disease characteristics in the actual clinical trial (BREEZE-AD PEDS phase III [29])

|  | **Placebo** | **BARI 1mg** | **BARI 2mg** | **BARI 4mg** |
| --- | --- | --- | --- | --- |
| n | 122 | 121 | 120 | 120 |
| Age (years) | 11.8 (4.0) | 12.4 (4.1) | 11.8 (3.7) | 11.9 (3.8) |
| vIGA-AD: 4, n (%) | 48 (39.3) | 45 (37.5) | 46 (38.3) | 45 (37.5) |
| Age at AD diagnosis (years) | 2.6 (3.6) | 2.6 (3.9) | 2.5 (3.5) | 3.9 (4.0) |
| Sex: female, n (%) | 64 (52.5) | 62 (51.2) | 63 (52.5) | 53 (44.2) |

[Note] Data are presented as mean (SD) unless otherwise indicated. BARI: baricitinib; AD: atopic dermatitis; vIGA-AD: validated Investigator Global Assessment.

In addition, the probability of the treatment allocation correction value $b_{0}$ in the true propensity score model and outcome event rate correction value $a_{0}$ in the model that generates outcome data are shown below. Also, coefficient value $b_{r}$ of index variable $X_{r}$ for the information on whether the data are RCT data or not in the true propensity score model is shown below.

Probability of treatment allocation correction value $b_{0}$ in the true propensity score model in the simulation setting based on parameter settings from actual clinical trial.

| **Rt: Rc: H** | **Scenario Ⅰ** | **Scenario Ⅱ** |
| --- | --- | --- |
| 1：1：2 | -12.54 | -12.54 |

[Note] R: RCT, H: Historical control, t: treatment group, c: control group.

Outcome event rate correction value $a_{0}$ in the model that generates outcome data in the simulation setting based on parameter settings from actual clinical trial.

| **Scenario Ⅰ** | | | | | | |
| --- | --- | --- | --- | --- | --- | --- |
| Outcome  event rate | Odds ratio | | | | | |
|  | 1.0 | 1.5 | 2.0 | 2.5 | 5.0 | 10.0 |
| 50% | -2.901 | -3.002 | -3.073 | -3.126 | -3.278 | -3.406 |
| 10% | -5.243 | -5.356 | -5.452 | -5.535 | -5.844 | -6.234 |
| 5% | -6.011 | -6.126 | -6.226 | -6.314 | -6.655 | -7.092 |
| **Scenario Ⅱ** | | | | | | |
| Outcome  event rate | Odds ratio | | | | | |
|  | 1.0 | 1.5 | 2.0 | 2.5 | 5.0 | 10.0 |
| 50% | -3.049 | -3.151 | -3.221 | -3.274 | -3.428 | -3.556 |
| 10% | -5.392 | -5.504 | -5.599 | -5.682 | -5.989 | -6.377 |
| 5% | -6.160 | -6.274 | -6.373 | -6.461 | -6.799 | -7.234 |

[Note] R: RCT, H: historical control, t: treatment group, c: control group.

Coefficient value $b_{r}$ of index variable $X_{r}$ for the information on whether the data are randomized controlled trial data or not in the true propensity score model in the simulation setting based on parameter settings from actual clinical trial.

| **Rt: Rc: H** | **Scenario Ⅱ** |
| --- | --- |
| 1：1：2 | 11.62 |

[Note] R: RCT, H: Historical control, t: treatment group, c: control group.
